# Clinical and Functional Outcomes of Community-Recruited Individuals at Clinical High-Risk for Psychosis: Results From the Youth Mental Health Risk and Resilience Study (YouR-Study)

**DOI:** 10.1093/schizbullopen/sgae029

**Published:** 2024-11-12

**Authors:** Kate Haining, Ruchika Gajwani, Joachim Gross, Andrew I Gumley, Stephen M Lawrie, Frauke Schultze-Lutter, Matthias Schwannauer, Peter J Uhlhaas

**Affiliations:** School of Psychology and Neuroscience, University of Glasgow, Glasgow, UK; School of Health and Wellbeing, University of Glasgow, Glasgow, UK; School of Psychology and Neuroscience, University of Glasgow, Glasgow, UK; Institute for Biomagnetism and Biosignalanalysis, University of Münster, Münster, Germany; School of Health and Wellbeing, University of Glasgow, Glasgow, UK; Department of Psychiatry, University of Edinburgh, Edinburgh, UK; Department of Psychiatry and Psychotherapy, Medical Faculty and University Hospital Düsseldorf, Heinrich-Heine-University, Düsseldorf, Germany; Department of Psychology, Faculty of Psychology, Airlangga University, Surabaya, Indonesia; University Hospital of Child and Adolescent Psychiatry and Psychotherapy, University of Bern, Bern, Switzerland; Department of Clinical Psychology, University of Edinburgh, Edinburgh, UK; School of Psychology and Neuroscience, University of Glasgow, Glasgow, UK; Department of Child and Adolescent Psychiatry, Charité Universitätsmedizin, Berlin, Germany

**Keywords:** clinical high-risk, psychosis, early detection and intervention, web screening, longitudinal outcomes

## Abstract

Clinical high-risk for psychosis (CHR-P) individuals are typically recruited from clinical services but the clinical and functional outcomes of community-recruited CHR-P individuals remain largely unclear. The Youth Mental Health Risk and Resilience Study (YouR-Study) obtained a community sample of CHR-P individuals through an online-screening approach and followed-up these individuals for a period of up to 3 years to determine transition rates, persistence of attenuated psychotic symptoms (APS) and functional outcomes. Baseline data were obtained from *n* = 144 CHR-P participants, *n* = 51 participants who met online cutoff criteria but not CHR-P criteria (CHR-Ns), and *n* = 58 healthy controls. Baseline assessments included clinical measures for assessing CHR-P status, including the Comprehensive Assessment of At-Risk Mental States (CAARMS) and the Schizophrenia Proneness Instrument, Adult version (SPI-A), as well as functioning and cognitive measures. CHR-P and CHR-N groups were followed-up. Results show that 12.1% of CHR-P individuals transitioned to psychosis over 3 years, with no transitions in the CHR-N group. Nearly 60% of CHR-P individuals experienced poor functional outcome (PFO) and over 40% experienced persistent APS. A combination of CAARMS/SPI-A criteria was associated with a higher likelihood of PFO, but not with transition to psychosis nor APS persistence. However, transition risk was generally higher among those meeting both CAARMS/SPI-A criteria (64.3%) vs CAARMS (28.6%) or SPI-A (7.1%) alone. In summary, community-recruited CHR-P individuals are characterized by similar clinical characteristics and longitudinal outcomes to those recruited from clinical services, emphasizing the need to widen the scope of early detection and intervention strategies.

## Introduction

Schizophrenia-spectrum and primary psychotic disorders have a peak age of onset during the transition from adolescence to adulthood^1^ and are preceded, in the majority of cases, by a clinical high-risk for psychosis (CHR-P) state lasting approximately 5–6 years.^2^ CHR-P individuals have heterogenous clinical presentations and outcomes, including attenuated positive and negative symptoms as well as basic symptoms (BS), affective disturbances, impaired functioning, and cognitive deficits.^3–5^

Approximately 25% of CHR-P individuals transition to psychosis within 3 years of follow-up.^6^ Baseline predictors associated with transition to psychosis include attenuated psychotic symptoms (APS), negative symptoms, poor functioning, verbal memory impairments, and certain electrophysiological markers.^7^ Recruitment pathways have also been shown to impact on transition rates, with community-recruitment strategies potentially diluting psychosis risk.^8^ Importantly, approximately 50% of CHR-P participants experience persistent APS^9^ as well as impairments in social and role functioning.^10,11^

CHR-P criteria used to identify CHR-P individuals include ultra-high risk (UHR) and BS criteria.^12,13^ Notably, UHR criteria were designed to detect the imminent risk of psychosis (ie, within the next 12 months) whereas BS criteria were designed to detect psychosis risk as early as possible in the development of the disorder.^2^ Symptomatic UHR criteria comprise APS and brief limited intermittent psychotic symptoms (BLIPS) while BS criteria comprise cognitive disturbances (COGDIS) and cognitive-perceptive BS (COPER), which primarily involve subjective cognitive disturbances. Importantly, individuals meeting both UHR (APS and/or BLIPS) and COGDIS criteria at baseline have a significantly higher risk of transition and a shorter time to transition than those meeting either criterion alone.^14^ In addition, the persistence rate of BS also appears to be higher than that of APS.^3^

Notably, current early detection strategies only detect 5%–12% of first-episode psychosis patients during their CHR-P stage,^15,16^ suggesting that a broader approach to identifying emerging psychosis may be needed to extend the benefits of early intervention. A large representative Swiss community study using gold-standard CHR-P assessments via telephone interviews found that 1.1% of respondents met APS, BLIPS, and/or COGDIS criteria, with a 3-year transition rate of 11.1%, indicating that the application of CHR-P criteria outside of clinical samples may be feasible.^17,18^

While it is not recommended to directly screen individuals in the community using gold-standard CHR-P assessment methods, risk enrichment strategies, such as prescreening questionnaires, may allow the extension of CHR-P detection.^19,20^ Using such an approach, the Youth Mental Health Risk and Resilience Study (YouR-Study)^21^ recently provided initial evidence for the feasibility of a web-based screening platform for detecting CHR-P individuals in the community.^22^

Previous publications by our group have reported baseline demographic, clinical, functioning, and cognitive data from a subset of YouR-Study participants.^22–27^ In the current article, we aim to provide a comprehensive overview of YouR-Study participants, encompassing baseline data and longitudinal outcomes over a 3-year follow-up period. Our primary goal was to investigate differences in baseline demographic, clinical, functional, and cognitive data between CHR-P subgroups (persistent vs non-persistent APS, poor vs good functional outcome [GFO], transition vs non-transition), to characterize longitudinal outcomes, as well as to examine the utility of UHR and BS criteria in predicting clinical and functional outcomes, including transition to psychosis. Specifically, we formulated the following hypotheses:

1. CHR-P subgroups will display greater impairments in clinical, functioning, cognitive, and questionnaire measures at baseline. These subgroups include individuals who later transition to psychosis, individuals with poor functional outcome (PFO), and those with APS persistence.
2. CHR-P individuals meeting both Comprehensive Assessment of At-Risk Mental States (CAARMS) and Schizophrenia Proneness Instrument, Adult version (SPI-A) criteria or alternatively APS and COGDIS criteria will have the greatest risk of poor clinical and functional outcomes, including transition to psychosis.
3. CHR-P participants will show significant improvements in clinical measures across follow-up yet continue to display persistent APS and poor functioning.

## Methods

### Participants

Participants were recruited between 2014 and 2019 as part of the MRC-funded YouR-Study^21^ which sought to identify neurobiological and psychological mechanisms and predictors of psychosis risk. Participants meeting CHR-P criteria were recruited through a web-based screening platform,^22^ comprising (1) the 16-item Prodromal Questionnaire (PQ-16)^28^ and (2) a 9-item scale of Perceptual and Cognitive Anomalies (PCA) for assessing BS. Participants with a cutoff score of 6 or more on the PQ-16 and/or a cutoff score of 3 or more on the PCA were invited for further clinical interviews. CHR-N participants also met the online-screening threshold, but did not meet CHR-P criteria during the clinical interviews. Healthy control (HC) participants were recruited from a volunteer database in Glasgow. Previous research has emphasized the need to include both healthy and psychiatric comparison groups in CHR-P studies in order to identify the specific risk factors associated with psychosis.^29^

Baseline data were available for *n* = 144 CHR-P participants (Glasgow site: 73.6%, Edinburgh site: 26.4%), *n* = 51 CHR-N participants (Glasgow site: 82.4%, Edinburgh site: 17.6%), and *n* = 58 HCs. One hundred and sixteen CHR-P participants (80.6%) and *n* = 30 CHR-N participants (58.8%) completed at least 1 follow-up assessment.

Ethical approval was obtained from the West of Scotland Research Ethics Service and the University of Glasgow. All participants provided written informed consent.

### Baseline Assessments

The positive scale of the CAARMS^12^ and the COGDIS and COPER items of the SPI-A^13^ were administered to establish CHR-P criteria. Participants were recruited into the CHR-P group if they met one or both SPI-A criteria (ie, COGDIS, COPER) and/or at least one of the following CAARMS criteria: (1) APS group (APS present in the last year without a decline in functioning); (2) genetic risk and functional deterioration group; and (3) BLIPS group.

While both COPER and COGDIS capture subjective cognitive disturbances (eg, thought blockages), COPER also includes subjective perceptual disturbances (eg, visual perception disturbances). To meet COGDIS criteria, participants were required to endorse at least 2 of 9 BS—5 of which are also included in the COPER criteria—with a frequency of at least “several times in a month or weekly” within the past 3 months. To meet COPER criteria, participants were required to report at least 1 of 10 BS as having first occurrence more than 12 months ago and a frequency of at least “several times in a month or weekly” within the past 3 months. Notably, for inclusion in the CAARMS APS group, the symptom(s) must have started or worsened in the past year—a criterion from the Structured Interview for Psychosis-risk Syndromes.^30^ Indeed, this criterion may exclude a large percentage of individuals who are no longer at risk of transition.^31^ At follow-up, this criterion remained, although participants could also qualify for the APS group if the symptom(s) reported at baseline/previous follow-up persisted.

All participant groups were assessed with the Global Assessment of Functioning (GAF) scale from the DSM-IV-TR, National Adult Reading Test (NART),^32^ Brief Assessment of Cognition in Schizophrenia (BACS),^33^ and 3 tasks from the Penn Computerized Neurocognitive Battery (CNB)^34^: The Continuous Performance Test, the N-Back Test, and the Emotion Recognition Task which provide measures of accuracy and response time for attention, working memory and emotion recognition, respectively.

All participants were also assessed with the Mini-International Neuropsychiatric Interview (MINI),^35^ Global Functioning: Social (GF: Social) and Role (GF: Role) scales,^36^ Premorbid Adjustment Scale (PAS),^37^ Inventory of Interpersonal Problems (IIP-32),^38^ International Positive and Negative Affect Schedule—Short Form (IPANAS-SF),^39^ Significant Others Scale (SOS),^40^ Social Interaction Anxiety Scale (SIAS),^41^ Adverse Childhood Experiences (ACEs) Questionnaire,^42^ Beliefs About Paranoia Scale (BAPS),^43^ Brief Core Schema Scale (BCSS),^44^ Psychosis Attachment Measure (PAM),^45^ and Rust Inventory of Schizotypal Cognitions (RISC).^46^ See supplementary table 1 for a full baseline assessment schedule per group.

All assessments were administered by trained research assistants and MSc/PhD level researchers. Inter-rater reliability of CHR-P status, determined by CAARMS and SPI-A ratings, was excellent (CAARMS: 92.0%; SPI-A: 95.7%).

### Follow-up Assessments

CHR-P and CHR-N participants were invited for follow-up assessments at 6, 12, 18, 24, 30, and 36 months (supplementary figures 1 and 2). The CAARMS, GAF, IIP-32, IPANAS-SF, and SOS were administered at all follow-up assessments. In addition, the GF: Social and GF: Role scales and the Structured Clinical Interview for DSM-IV (SCID)^47^ were included at 6, 12, and 24 months, while the SPI-A was included at 24 and 36 months.

APS persistence was operationalized as meeting APS criteria at both baseline and follow-up; whilst APS non-persistence was operationalized as meeting APS criteria at baseline but not at follow-up. Meanwhile, BS persistence was operationalized as meeting BS criteria at both baseline and 24-month follow-up. In addition, PFO and GFO were defined as GAF scores <65 and ≥65, respectively.^48,49^ Transitions to psychosis were defined according to CAARMS criteria and subsequently followed-up with the SCID to establish the specific psychosis diagnoses. Once an individual transitioned, they were not invited for further follow-up assessments.

### Statistical Analysis

Data were analyzed using SPSS version 28 and R version 4.1.3.^50^ Overall, 1.9% of the data (490 of 25 318 values) were missing from completed assessments and imputed by Bayesian imputation (see supplementary methods for the categorization of current MINI comorbidities and calculations related to symptom severity/distress, cognitive data, and questionnaire data). At baseline, between-group differences for more than 2 groups were analyzed using 1-way ANOVAs, Kruskal-Wallis *H* tests, and Pearson’s chi-squared or Fisher-Freeman-Halton tests, followed by appropriate Bonferroni-corrected post hoc tests if required. Between-group differences for 2 groups were analyzed using Welch’s *t* tests, Mann-Whitney *U* tests, and Pearson’s chi-squared or Fisher’s exact tests.

For longitudinal analyses, follow-up data were collapsed into the following timepoints—6–12 and 18–24 months—such that the last available follow-up data was utilized at each timepoint. Note that 30–36 months was not included here to preserve the sample size. First, we focused on primary variables which were available at all follow-up assessments ie, CAARMS, GAF, IIP-32, IPANAS-SF, and SOS. Second, we examined secondary variables which were not available at all follow-up assessments ie, SPI-A, MINI, SCID, GF: Social, and GF: Role. Where data were available for baseline and 2 timepoints, within-group differences were analyzed using repeated measures ANOVAs, Friedman tests, and Cochran’s *Q* tests. Post hoc analyses were conducted following significant results with a Bonferroni correction applied, resulting in a significance level set at *P* < .017 for each variable. Where data were available for baseline and 1 timepoint or where post hoc analyses were required, group differences were analyzed using paired *t* tests, Wilcoxon signed-rank tests, and McNemar’s tests.

Kaplan-Meier survival analysis was employed to calculate the cumulative hazard rates associated with transition to psychosis among CHR-P individuals with follow-up data. First, we compared CHR-P individuals meeting CAARMS criteria only, SPI-A criteria only, or both criteria combined at baseline (general risk criteria). Second, we compared CHR-P who did and did not meet both APS and COGDIS criteria at baseline (specific risk criteria). Survival curves were compared using the Tarone-Ware test. The survival time ended with transition to psychosis or, for censored cases, with the end of the follow-up period (36 months) or withdrawal from the study. Similarly, we examined whether general and/or specific risk criteria could predict APS persistence and/or PFO at 12 months using binary logistic regression analyses.

## Results

### Baseline Demographic and Clinical Data

CHR-P participants were significantly younger than HC participants (table 1). CHR-P participants also had significantly fewer years of education than HC participants and females were over-represented in all groups (CHR-P = 72.2%, CHR-N = 66.7%, HC = 70.7%).

**Table 1.** Demographic, Clinical, Functional, and Cognitive Characteristics of CHR-P (*n* = 144), CHR-N (*n* = 51), and HC (*n* = 58) Participants at Baseline

	CHR-P (1) (*n* = 144)	CHR-N (2) (*n* = 51)	HC (3) (*n* = 58)	*P*	Effect Size^e^	Post Hoc Test
Demographic, clinical, and functional data						
Age (y), mean (SD)	21.63 (4.14)	22.88 (4.74)	22.62 (3.38)	.025	η^2^_p_ = 0.029	1 < 3
Sex, female *n* (%)	104 (72.2)	34 (66.7)	41 (70.7)	.755	*V* = 0.047	—
Education (y), mean (SD)	15.41 (2.92)	16.45 (3.50)	16.55 (2.93)	.020	η^2^_p_ = 0.030	1 < 3
Current medication, *n* (%)						
Antidepressant use	35 (24.3)	9 (17.6)	0 (0)	<.001	*V* = 0.259	1, 2 > 3
Anxiolytic use	15 (10.4)	3 (5.9)	0 (0)	.014	*V* = 0.166	1 > 3
Antipsychotic use	3 (2.1)	0 (0)	0 (0)	.579	*V* = 0.095	—
Current MINI comorbidity, *n* (%)^a^						
Anxiety disorder	103 (74.6)	22 (46.8)	0 (0)	<.001	*V* = 0.614	1 > 2 > 3
Mood disorder	78 (56.5)	11 (23.4)	0 (0)	<.001	*V* = 0.499	1 > 2 > 3
Alcohol abuse/dependence	43 (31.2)	11 (23.4)	0 (0)	<.001	*V* = 0.308	1, 2 > 3
Drug abuse/dependence	22 (15.9)	3 (6.4)	0 (0)	.002	*V* = 0.224	1 > 3
Eating disorder	13 (9.4)	1 (2.1)	0 (0)	.011	*V* = 0.182	1 > 3
Current psychological therapy, *n* (%)	26 (18.1)	5 (9.8)	0 (0)	.002	*V* = 0.226	1, 2 > 3
CAARMS severity, median (range)	28 (0–74)	5 (0–24)	0 (0–12)	<.001	η^2^_p_ = 0.387	1 > 2 > 3
CAARMS mean distress, median (range)^b^	52.5 (0–100)	30 (0–75)	0 (0–100)	<.001	η^2^_p_ = 0.087	1 > 2, 3
CAARMS max distress, median (range)^b^	80 (0–100)	50 (0–90)	0 (0–100)	<.001	η^2^_p_ = 0.132	1 > 2, 3
SPI-A severity, median (range)	7 (0–74)	0 (0–10)	0 (0–2)	<.001	η^2^_p_ = 0.338	1 > 2, 3
SPI-A mean distress, median (range)^c^	33 (0–93)	20 (0–80)	0 (0–40)	.021	η^2^_p_ = 0.048	—
SPI-A max distress, median (range)^c^	50 (0–100)	20 (0–90)	0 (0–40)	.001	η^2^_p_ = 0.081	1 > 3
GAF, median (range)	58 (21–95)	70 (43–94)	88 (67–97)	<.001	η^2^_p_ = 0.330	1 < 2 < 3
PAS, median (range)^a^	1.14 (0–3)	0.86 (0–4)	0.43 (0–2)	<.001	η^2^_p_ = 0.178	1, 2 > 3
GF: Social current, median (range)^a^	8 (3–10)	8 (6–9)	9 (8–10)	<.001	η^2^_p_ = 0.228	1 < 2 < 3
GF: Role current, median (range)^a^	8 (3–9)	8 (5–9)	9 (5–9)	<.001	η^2^_p_ = 0.202	1 < 2 < 3
Cognitive data, mean (SD)^d^						
Premorbid IQ	110.14 (7.06)	110.06 (7.10)	110.47 (6.88)	.948	ω² = 0.008	—
Verbal memory	−0.09 (1.17)	0.13 (1.04)	0.05 (1.01)	.475	ω² = 0.002	—
Working memory	−0.01 (1.33)	0.25 (1.04)	0.02 (1.01)	.461	η^2^_p_ = 0.007	—
Motor speed	−0.62 (1.08)	−0.33 (0.90)	0 (0.98)	<.001	η^2^_p_ = 0.061	1 < 3
Verbal fluency	−0.10 (0.93)	−0.17 (0.84)	0 (1.02)	.930	η^2^_p_ = 0.001	—
Attention and processing speed	−0.40 (1.12)	0.12 (1.21)	0 (1.00)	.004	η^2^_p_ = 0.046	1 < 2, 3
Executive function	0.04 (1.18)	−0.02 (1.14)	−0.01 (0.97)	.863	η^2^_p_ = 0.001	—
BACS total	−0.44 (1.54)	−0.01 (1.30)	0.02 (1.00)	.039	ω² = 0.016	1 < 3
Emotion recognition efficiency	−0.30 (0.91)	−0.13 (0.84)	0 (0.68)	.167	η^2^_p_ = 0.015	—
Working memory efficiency	−0.08 (0.81)	0.01 (0.80)	0 (0.76)	.654	η^2^_p_ = 0.003	—
Attention efficiency	−0.08 (0.88)	0.08 (0.82)	0 (0.63)	.321	η^2^_p_ = 0.010	—

*Note:* BACS, Brief Assessment of Cognition in Schizophrenia; CAARMS, Comprehensive Assessment of At-Risk Mental States; CHR-N, clinical high-risk-negative; CHR-P, clinical high-risk for psychosis; GAF, Global Assessment of Functioning; GF, Global Functioning; HC, healthy control; MINI, Mini-International Neuropsychiatric Interview; PAS, Premorbid Adjustment Scale; SPI-A, Schizophrenia Proneness Instrument, Adult version.

^a^Refers to *n* = 138 CHR-P participants and *n* = 47 CHR-N participants ie, those with Visit 2 data.

^b^Refers to *n* = 139 CHR-P participants, *n* = 35 CHR-N participants and *n* = 7 HC participants ie, those with CAARMS severity >0.

^c^Refers to *n* = 127 CHR-P participants, *n* = 23 CHR-N participants and *n* = 6 HC participants ie, those with SPI-A severity >0.

^d^Refers to *n* = 135 CHR-P participants, *n* = 47 CHR-N participants and *n* = 55 HC participants ie, those with cognitive data.

^e^Effect sizes were eta squared (η^2^_p_) for Kruskal-Wallis *H* tests (small effect = 0.01, medium effect = 0.06, large effect = 0.14), omega squared (ω²) for 1-way ANOVAs (small effect = 0.01, medium effect = 0.06, large effect = 0.14), and Cramer’s *V* for Pearson’s chi-squared or Fisher-Freeman-Halton exact tests (small effect = 0.1, medium effect = 0.3, large effect = 0.5).

Among the CHR-P group, *n* = 39 participants (27.1%) met CAARMS criteria, *n* = 36 (25.0%) met SPI-A criteria, and *n* = 69 (47.9%) met both CAARMS and SPI-A criteria (see supplementary results, Diagnostic categories). The CHR-P group had greater CAARMS severity and distress, greater SPI-A severity, higher likelihood of comorbid mood and anxiety disorders, lower social and role functioning, and poorer global functioning compared with CHR-N and HC groups (table 1, supplementary table 2). In addition, significantly more CHR-P (18.1%) and CHR-N (10.6%) participants were classified into the high suicide risk category relative to HC participants (0%).

Notably, the *n* = 3 CHR-P participants on antipsychotic medications were taking dosages below the level generally recommended for antipsychotic action. None of the CHR-P participants were in contact with CHR-P or psychosis services at baseline.

### Baseline Cognitive Data

CHR-P individuals had significantly poorer attention and processing speed than both CHR-N and HC individuals (table 1).

### Baseline Questionnaire Data

CHR-P participants displayed fewer positive evaluations of self (BCSS) as well as greater negative affect (IPANAS-SF), interpersonal problems (IIP-32), dissatisfaction with practical support (SOS), social interaction anxiety (SIAS), negative beliefs about paranoia, beliefs about paranoia as a survival strategy and normalizing beliefs (BAPS), negative evaluations of self and others (BCSS), anxious and avoidant attachment (PAM) and schizotypal cognitions (RISC) compared with CHR-N and HC participants (supplementary table 2).

### Longitudinal Course

CHR-P individuals who attended at least 1 follow-up assessment and CHR-P individuals who did not attend follow-up assessments were relatively similar in terms of baseline characteristics, although the latter group had significantly greater RISC schizotypal cognitions (supplementary tables 3 and 4).

**Fig. 1.**
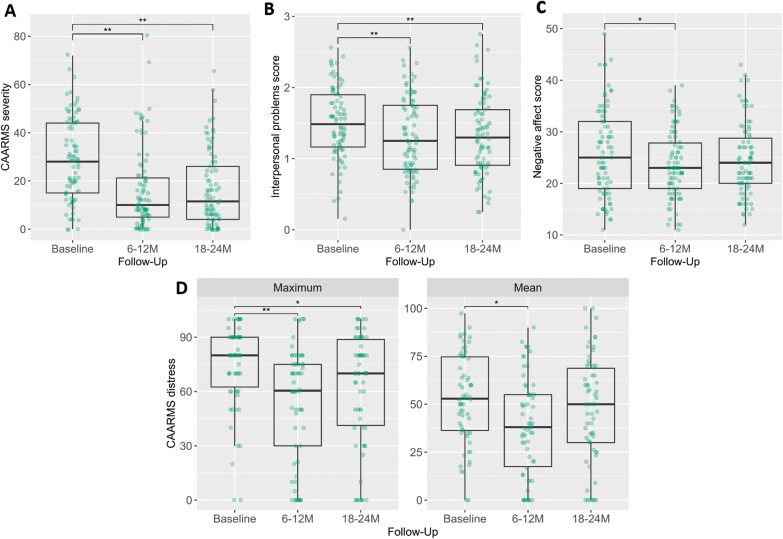
Changes in (A) CAARMS severity (*n* = 84); (B) IIP-32 interpersonal problems (*n* = 82); (C) IPANAS-SF negative affect (*n* = 82); and (D) CAARMS maximum (*n* = 62) and mean (*n* = 62) distress over follow-up among CHR-P individuals. Dots represent individual participants. *P < .017 (alpha level calculated using Bonferroni correction for 3 comparisons), **P < .001. *Note*: CAARMS, Comprehensive Assessment of At-Risk Mental States; CHR-P, clinical high-risk for psychosis; IIP-32, Inventory of Interpersonal Problems; IPANAS-SF, International Positive and Negative Affect Schedule—Short Form.

In the first step, we focused on a subgroup of CHR-P participants (*n* = 84) who completed primary measures at baseline, 6–12 months follow-up, and 18–24 months follow-up (figure 1, table 2). We found significant reductions in CAARMS severity, the proportion of individuals meeting APS criteria only, CAARMS maximum distress and interpersonal problems (IIP-32) between baseline and 6–12 months follow-up and between baseline and 18–24 months follow-up. In addition, there was a significant reduction in CAARMS mean distress and negative affect (IPANAS-SF) between baseline and 6–12 months follow-up. Post hoc comparisons for practical support satisfaction (SOS) did not survive Bonferroni correction.

**Table 2.** Subgroup Analysis of Primary Variables Over Follow-up Among CHR-P Individuals (*n* = 84)

	Baseline	6–12 mo	18–24 mo	*P*	1 vs 2		1 vs 3		2 vs 3	
	(1)	(2)	(3)		*P*	ES^d^	*P*	ES^d^	*P*	ES^d^
Clinical and functional data										
CAARMS severity, median (range)	28 (0–72)	10 (0–80)	11.5 (0–66)	<.001	<.001	*r* = .640	<.001	*r* = .667	.582	*r* = .060
CAARMS mean distress, median (range)^a^	52.9 (0–97.5)	38 (0–90)	50 (0–100)	.036	.001	*r* = .410	.121	*r* = .197	.099	*r* = .209
CAARMS max distress, median (range)^a^	80 (0–100)	60.5 (0–100)	70 (0–100)	.003	<.001	*r* = .488	.009	*r* = .330	.162	*r* = .178
UHR criteria met, *n* (%)^b^										
APS only	59 (70.2)	34 (40.5)	30 (35.7)	<.001	<.001	*g* = 0.357	<.001	*g* = 0.354	.596	*g* = 0.063
BLIPS only	0 (0)	1 (1.2)	0 (0)	1.000						
GRFD only	3 (3.6)	0 (0)	0 (0)	.111						
APS persistence, n (%)		29 (34.5)	24 (28.6)	.424						
GAF, median (range)	59.5 (21–91)	62 (21–87)	58 (21–88)	.259						
Questionnaire data^c^										
Positive affect, mean (SD)	25.75 (7.16)	26.82 (9.32)	26.25 (7.87)	.459						
Negative affect, mean (SD)	25.85 (8.62)	23.61 (6.52)	24.50 (7.10)	.040	.015	*d* = 0.276	.167	*d* = 0.154	.216	*d* = 0.138
Interpersonal problems, mean (SD)	1.50 (0.51)	1.32 (0.56)	1.32 (0.56)	<.001	<.001	*d* = 0.411	<.001	*d* = 0.381	.869	*d* = 0.018
Practical support satisfaction, mean (SD)	0.99 (0.69)	0.79 (0.67)	0.79 (0.65)	.035	.037	*d* = 0.234	.032	*d* = 0.242	.944	*d* = 0.008
Emotional support satisfaction, mean (SD)	1.08 (0.77)	0.90 (0.69)	1.00 (0.79)	.102						

*Note*: APS, attenuated psychotic symptoms; BLIPS, brief limited intermittent psychotic symptoms; CAARMS, Comprehensive Assessment of At-Risk Mental States; CHR-P, clinical high-risk for psychosis; GAF, Global Assessment of Functioning; GRFD, genetic risk, and functional deterioration; UHR, ultra-high risk.

^a^Refers to *n* = 62 CHR-P participants ie, those with a CAARMS severity score >0 across all timepoints.

^b^UHR criteria for APS, BLIPS, and GRFD are not mutually exclusive.

^c^Refers to *n* = 82 CHR-P participants ie, individuals with questionnaire data across all timepoints.

^d^Effect sizes were Rosenthal’s *r* for Wilcoxon signed-rank tests (small effect = 0.1, medium effect = 0.3, large effect = 0.5), Cohen’s *g* for McNemar’s test (small effect = 0.05, medium effect = 0.15, large effect = 0.25), and Cohen’s *d* for paired *t* tests (small effect = 0.2, medium effect = 0.5, large effect = 0.8).

Second, we focused on a smaller subgroup of CHR-P participants (*n* = 64) who completed secondary measures at baseline, 6–12 months follow-up, and 24 months follow-up (supplementary table 5). We found a significant reduction in the proportion of CHR-P individuals meeting COPER criteria only between baseline and 24 months follow-up. The longitudinal analyses of primary and secondary variables were repeated following the removal of 7 CHR-P participants who transitioned to psychosis (supplementary tables 6 and 7). These analyses confirmed the above results, except for the reduction in negative affect (IPANAS-SF) between baseline and 6–12 months. For the longitudinal analyses of primary and secondary variables in the CHR-N group, see supplementary material (supplementary results, supplementary tables 8 and 9).

### Clinical Outcome

Of the *n* = 116 CHR-P individuals with follow-up data, *n* = 14 transitioned to psychosis (transition risk: 12.1%). The median time to transition was 15 months (range = 6–36 months; supplementary figure 1). Notably, we did not identify any psychosis transitions in the CHR-N sample although *n* = 8 participants met APS criteria at 1 or more follow-up assessments (supplementary figure 2).

At baseline, CHR-P individuals who transitioned to psychosis had significantly poorer global, social, and premorbid functioning, higher likelihood of comorbid mood disorder and alcohol abuse/dependence, fewer positive evaluations of self and others (BCSS) and higher levels of avoidant attachment (PAM) than those who did not transition (supplementary tables 10 and 11).

**Fig. 2.**
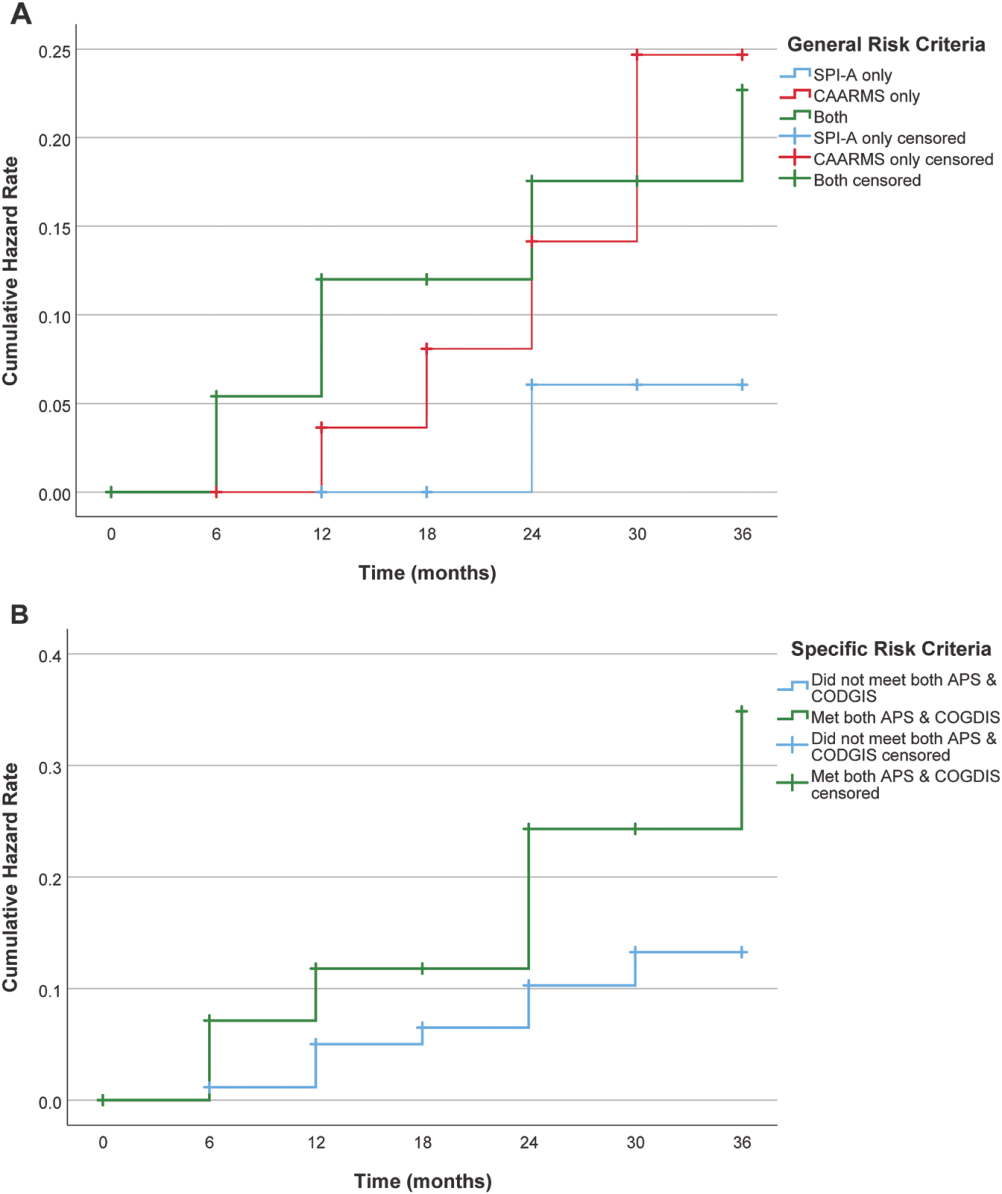
Kaplan-Meier analysis for (A) CHR-P individuals meeting CAARMS criteria only, SPI-A criteria only, or both at baseline (*n* = 116) and (B) CHR-P individuals who did and did not meet both APS and COGDIS criteria at baseline (*n* = 116). Vertical lines indicate censoring events, while steps represent transition events in the CHR-P population over the follow-up period. *Note*: APS, attenuated psychotic symptoms; CAARMS, Comprehensive Assessment of At-Risk Mental States; CHR-P, clinical high-risk for psychosis; COGDIS, Cognitive Disturbances; SPI-A, Schizophrenia Proneness Instrument, Adult version.

There were no significant differences in survival curves for general CHR-P risk criteria, χ^2^ (2, *n* = 116) = 2.738, *P* = .254 (figure 2A). Notably, *n* = 29 of *n* = 116 (25.0%) CHR-P individuals met both APS and COGDIS criteria at baseline, 6 of whom later transitioned to psychosis (20.7%). Although there was no significant difference in survival curves for specific risk criteria (figure 2B), we did identify a possible trend for higher risk of transition in CHR-P who did, vs did not, meet both APS and COGDIS criteria at baseline, χ^2^ (1, *n* = 116) = 2.794, *P* = .095 (see supplementary table 12 for cumulative hazard rates for transition at different follow-up assessments).

### Functional Outcome and APS Persistence

At 12 months follow-up, *n* = 67 (57.8%) CHR-P participants had PFO while *n* = 49 (42.2%) had GFO. At baseline, CHR-P participants with PFO were impaired on several clinical, functional, cognitive, and questionnaire measures. For example, individuals with PFO had greater antidepressant use, higher CAARMS severity and distress, and greater likelihood of high suicide risk, comorbid anxiety disorders, and comorbid mood disorders (table 3, supplementary table 13). In addition, CHR-P participants with PFO had poorer emotion recognition efficiency and attention efficiency as well as greater ACEs, negative evaluations of self and others (BCSS), levels of avoidant attachment (PAM), and schizotypal cognitions (RISC) than those with GFO.

**Table 3.** Demographic, Clinical, Functional, and Cognitive Characteristics of PFO (*n* = 67) vs GFO (*n* = 49) Participants and Persistent APS (*n* = 38) vs Non-persistent APS (*n* = 47) Participants at Baseline

	PFO (*n* = 67)	GFO (*n* = 49)	*P*	Effect Size^d^	Persistent APS (*n* = 38)	Non-persistent APS (*n* = 47)	*P*	Effect Size^d^
Demographic, clinical and functional data								
Age (y), mean (SD)	21.72 (4.70)	21.92 (3.59)	.259	*r* = .105	22.47 (4.88)	21.51 (4.19)	.434	*r* = .085
Sex, female *n* (%)	48 (71.6)	34 (69.4)	.792	ϕ = 0.024	27 (71.1)	35 (74.5)	.725	ϕ = 0.038
Education (y), mean (SD)	15.43 (3.18)	15.67 (2.95)	.539	*r* = .057	15.61 (3.48)	15.66 (3.06)	.943	*r* = .008
Current medication, *n* (%)								
Antidepressant use	21 (31.3)	7 (14.3)	.034	ϕ = 0.197	10 (26.3)	14 (29.8)	.724	ϕ = 0.038
Anxiolytic use	7 (10.4)	5 (10.2)	.966	ϕ = 0.004	3 (7.9)	7 (14.9)	.501	ϕ = 0.108
Antipsychotic use	1 (1.5)	0 (0)	1.000	ϕ = 0.080	1 (2.6)	0 (0)	.447	ϕ = 0.121
Current MINI comorbidity, *n* (%)								
Anxiety disorder	57 (85.1)	29 (59.2)	.002	ϕ = 0.292	30 (78.9)	36 (76.6)	.796	ϕ = 0.028
Mood disorder	42 (62.7)	20 (40.8)	.020	ϕ = 0.217	20 (52.6)	27 (57.4)	.657	ϕ = 0.048
Alcohol abuse/dependence	21 (31.3)	13 (26.5)	.574	ϕ = 0.052	10 (26.3)	15 (31.9)	.573	ϕ = 0.061
Drug abuse/dependence	9 (13.4)	8 (16.3)	.663	ϕ = 0.040	5 (13.2)	8 (17.0)	.623	ϕ = 0.053
Eating disorder	5 (7.5)	4 (8.2)	1.000	ϕ = 0.013	5 (13.2)	4 (8.5)	.505	ϕ = 0.075
Current psychological therapy, *n* (%)	13 (19.4)	7 (14.3)	.471	ϕ = 0.067	5 (13.2)	8 (17.0)	.623	ϕ = 0.053
CHR-P criteria met, *n* (%)								
CAARMS only	17 (25.4)	14 (28.6)	.701	ϕ = 0.036	10 (26.3)	20 (42.6)	.119	ϕ = 0.169
SPI-A only	10 (14.9)	18 (36.7)	.007	ϕ = 0.252	0 (0)	0 (0)	—	—
CAARMS and SPI-A	40 (59.7)	17 (34.7)	.008	ϕ = 0.247	28 (73.7)	27 (57.4)	.119	ϕ = 0.169
CAARMS severity, median (range)	31 (0–74)	21 (0–72)	.021	*r* = .215	42.5 (12–74)	30 (9–72)	.064	*r* = .201
CAARMS mean distress, median (range)	58.5 (0–100)^a^	41.3 (0–97.5)^a^	.008	*r* = .252	60.1 (17.5–97.5)	50 (0–100)	.406	*r* = .090
CAARMS max distress, median (range)	80 (0–100)^a^	70 (0–100)^a^	.003	*r* = .286	85 (30–100)	80 (0–100)	.301	*r* = .112
SPI-A severity, median (range)	9 (0–74)	7 (0–39)	.395	*r* = .079	7 (0–74)	6 (0–39)	.355	*r* = .100
SPI-A mean distress, median (range)	32 (0–70)^b^	33.3 (0–93)^b^	.472	*r* = .071	43.4 (0–93)^c^	28.8 (0–75)^c^	.016	*r* = .280
SPI-A max distress, median (range)	50 (0–100)^b^	50 (0–100)^b^	.895	*r* = .013	67.5 (0–100)^c^	48 (0–100)^c^	.017	*r* = .277
GAF, median (range)	52 (21–80)	64 (40–91)	< .001	*r* = .475	55 (21–91)	55 (21–87)	.856	*r* = .020
PAS, median (range)	1.36 (0–3)	0.93 (0–2)	.002	*r* = .286	1.23 (0–3)	1.29 (0–3)	.661	*r* = .048
GF: Social current, median (range)	7 (3–9)	8 (6–10)	< .001	*r* = .382	8 (3–9)	7 (5–9)	.402	*r* = .091
GF: Role current, median (range)	7 (3–9)	8 (5–9)	< .001	*r* = .365	7 (5–9)	7 (4–9)	.425	*r* = .086
Cognitive data, mean (SD)								
Premorbid IQ	110.06 (7.23)	111.54 (5.93)	.228	*d* = 0.221	110.46 (7.83)	109.85 (6.19)	.699	*d* = 0.087
Verbal memory	−0.15 (1.14)	0.08 (1.14)	.275	*d* = 0.206	−0.13 (1.07)	−0.04 (1.10)	.703	*d* = 0.083
Working memory	−0.04 (1.49)	−0.02 (1.16)	.718	*r* = .034	−0.25 (1.48)	−0.12 (1.43)	.702	*d* = 0.084
Motor speed	−0.79 (1.13)	−0.53 (1.05)	.202	*d* = 0.239	−0.78 (1.14)	−0.69 (0.93)	.701	*d* = 0.086
Verbal fluency	−0.16 (1.00)	0.01 (0.74)	.286	*r* = .099	0.05 (1.10)	−0.16 (0.84)	.336	*d* = 0.217
Attention and processing speed	−0.51 (1.04)	−0.21 (1.20)	.179	*r* = .125	−0.31 (0.95)	−0.55 (1.08)	.290	*d* = 0.229
Executive function	−0.07 (0.95)	0.11 (1.27)	.087	*r* = .159	0.04 (0.77)	0 (1.27)	.660	*r* = .048
BACS total	−0.64 (1.63)	−0.21 (1.35)	.125	*d* = 0.282	−0.51 (1.50)	−0.58 (1.58)	.824	*d* = 0.048
Emotion recognition efficiency	−0.42 (0.90)	−0.12 (0.96)	.019	*r* = .218	−0.26 (1.05)	−0.33 (0.87)	.474	*r* = .078
Working memory efficiency	−0.16 (0.88)	0.01 (0.74)	.334	*r* = .090	−0.12 (0.87)	−0.08 (0.77)	.912	*r* = .012
Attention efficiency	−0.33 (0.99)	0.21 (0.59)	.004	*r* = .268	0.02 (0.83)	−0.14 (0.86)	.372	*r* = .097

*Note*: APS, attenuated psychotic symptoms; BACS, Brief Assessment of Cognition in Schizophrenia; CAARMS, Comprehensive Assessment of At-Risk Mental States; GAF, Global Assessment of Functioning; GF, Global Functioning; GFO, good functional outcome; HC, healthy control; MINI, Mini-International Neuropsychiatric Interview; PAS, Premorbid Adjustment Scale; PFO, poor functional outcome; SCID, Structured Clinical Interview for DSM-IV; SPI-A, Schizophrenia Proneness Instrument, Adult version.

^a^Refers to *n* = 112 CHR-P participants (PFO = 64, GFO = 48) ie, those with CAARMS severity >0.

^b^Refers to *n* = 104 CHR-P participants (PFO = 59, GFO = 45) ie, those with SPI-A severity >0.

^c^Refers to *n* = 74 CHR-P participants (Persistent APS = 34, Non-persistent APS = 40) ie, those with SPI-A severity >0.

^d^Effect sizes were Rosenthal’s *r* for Mann-Whitney *U* tests (small effect = 0.1, medium effect = 0.3, large effect = 0.5), Cohen’s *d* for Welch’s *t* tests (small effect = 0.2, medium effect = 0.5, large effect = 0.8) and Phi (ϕ) for Pearson’s chi-squared or Fisher’s exact tests (small effect = 0.1, medium effect = 0.3, large effect = 0.5).

A logistic regression model with the presence/absence of PFO at 12-month follow-up as the outcome variable and general risk criteria met at baseline (ie, met CAARMS, met SPI-A, met both) as the predictor variable was statistically significant, χ^2^ (2, *n* = 116) = 9.36, *P* = .009, *R*^2^_N_ = 0.104. Specifically, participants who met both CAARMS and SPI-A criteria at baseline were over 4 times more likely to experience PFO at 12-month follow-up compared with those who met SPI-A only criteria at baseline (*P* = .003, odds ratio = 4.24; 95% CI = 1.62, 11.05). However, a logistic regression model with the presence/absence of PFO at 12-month follow-up as the outcome variable and specific risk criteria met at baseline (ie, met APS and COGDIS, did not meet APS and COGDIS) as the predictor variable was not statistically significant, χ^2^ (1, *n* = 116) = 2.04, *P* = .153, *R*^2^_N_ = 0.023.

At 12 months follow-up, *n* = 38 (44.7%) CHR-P participants had APS persistence while *n* = 47 (55.3%) had APS non-persistence. At baseline, CHR-P participants with APS persistence had significantly greater SPI-A distress and a higher likelihood of comorbid OCD (table 3, supplementary table 13).

A logistic regression model with the presence/absence of APS persistence at 12-month follow-up as the outcome variable and general risk criteria met at baseline as the predictor variable was not statistically significant, χ^2^ (1, *n* = 85) = 2.46, *P* = .117, *R*^2^_N_ = 0.038. Notably, general risk criteria in this analysis only referred to individuals meeting CAARMS only vs both CAARMS and SPI-A criteria due to the operationalization of APS persistence. Similarly, a logistic regression model with the presence/absence of APS persistence at 12-month follow-up as the outcome variable and specific risk criteria met at baseline as the predictor variable was not statistically significant, χ^2^ (1, *n* = 85) = 0.23, *P* = .634, *R*^2^_N_ = 0.004.

## Discussion

Research investigating CHR-P individuals has mostly focused on recruitment from clinical services and studies on community-recruited CHR-P individuals are rare.^17,18^ Hence, it is unclear whether community-recruited CHR-P individuals are characterized by similar baseline characteristics and longitudinal outcomes as CHR-P individuals recruited through clinical pathways. Our comprehensive review of the YouR-Study suggests that community-recruited CHR-P individuals share several key characteristics and outcomes with those recruited via clinical pathways, including transition risk, PFO, and APS persistence.

Community-recruited CHR-P individuals of the YouR-Study had an overall 3-year transition rate of 12.1%, which is in line with previous findings from a large representative Swiss community study.^17^ Also, we did not identify any psychosis transitions in the CHR-N sample. Although the transition rate is lower when compared with the recent meta-analytical estimate of 25% within 3 years,^6^ it does align with previous research focusing on CHR-P participants recruited via clinical pathways.^51,52^ Lower transition rates in our sample, however, likely reflect a dilution of psychosis risk due to wider community-recruitment as well as the considerable drop-out rate during follow-up. Notably, our findings also emphasize the substantial risk of adverse outcomes beyond transition to psychosis whereby 57.8% of CHR-P participants had PFO and 44.7% had APS persistence at 12-month follow-up, in line with previous findings.^9–11^

Furthermore, we found that specific subgroups within the CHR-P group, including individuals who transitioned to psychosis, individuals with APS persistence and individuals with PFO, displayed greater impairments in clinical, functioning, cognitive, and/or questionnaire measures at baseline. Importantly, CHR-P individuals with PFO exhibited cognitive deficits at baseline, consistent with previous findings linking cognitive impairments in emotion recognition and attention with PFO.^10,48^

Regarding clinical trajectory, we found a baseline to follow-up improvement in CAARMS symptom severity and distress, interpersonal problems and negative affect, and a reduction in the proportion of individuals meeting COPER criteria only and APS criteria only. Overall, these findings are consistent with data from a meta-analysis by Salazar de Pablo et al^9^ which reported a baseline to follow-up improvement in APS among individuals who did not transition to psychosis. Interestingly, however, this meta-analysis also reported an improvement in functioning which was not evident in our sample. Additionally, in line with data from previous studies in clinical samples,^3^ BS tended to be more persistent than APS in the current study.

Overall, our findings are in agreement with previous research in help-seeking clinical CHR-P samples.^3,9–11,48^ Notably, in the current study, only a small percentage of CHR-P individuals received psychological therapy and the number taking antidepressants and/or anxiolytics was relatively low considering the number of individuals presenting with depression and/or anxiety. As such, our results indicate that community-recruited CHR-P individuals represent a vulnerable group who would likely benefit from early intervention approaches and routine monitoring.

In terms of risk criteria, neither general nor specific risk criteria predicted transition to psychosis. These findings are in agreement with Youn et al^53^ who found that UHR individuals meeting COGDIS criteria did not have a greater risk of transition than those who met UHR criteria alone. In contrast, earlier research by Schultze-Lutter et al^14^ reported that combined risk identification approaches were shown to yield greater accuracy in identifying individuals at the highest risk of psychosis. One reason for the different findings may be the limited transition rates in both our study and the Youn et al^53^ study given that Schultze-Lutter et al^14^ reported a transition rate of 32.9% while the current study and Youn et al^53^ reported lower transition rates of 12.1% and 13%, respectively. Interestingly, however, CHR-P individuals who met both CAARMS and SPI-A criteria at baseline were over 4 times more likely to experience PFO at 12-month follow-up than those who met SPI-A only criteria at baseline (odds ratio = 4.24), suggesting that combined risk identification approaches could help to signal risk of adverse outcomes beyond transition to psychosis. However, neither general nor specific risk criteria demonstrated effectiveness in predicting APS persistence at 12-month follow-up, in contrast to the previous findings by Youn et al.^53^

### Strengths and Limitations

Next to the strengths of our study, in particular, the focus on community-recruited CHR-P participants, the comprehensive assessments (including both UHR and BS criteria), and the regular follow-up assessments over a 3-year period, some limitations must also be considered. First, 19.4% of CHR-P participants did not complete any follow-up assessments and, of those with follow-up data, 27.6% did not have sufficient data to be included in the longitudinal analysis. However, our dropout rates are consistent with those reported in previous clinical studies.^9^ Second, most of our sample were students engaged in college or university-level education (CHR-P = 86.1%, CHR-N = 78.4%, HC = 86.2%) and females were over-represented in all groups. Third, in terms of measurement issues, negative symptoms were not assessed and the GAF scale—used to define GFO and PFO—confounds functioning with symptom severity. Moreover, we did not routinely collect information on medication use or engagement in psychological therapy at follow-up.

## Conclusions

Our comprehensive overview of YouR-Study data provides novel evidence on clinical and functional outcomes and predictors in a sample of community recruited CHR-P individuals. Specifically, our data show substantial transition risk (12.1%) as well as persistent adverse outcomes in community-recruited CHR-P individuals. Indeed, despite demonstrating improvements in clinical symptoms over time, over 40% of CHR-P individuals experienced APS persistence and nearly 60% experienced PFO at 12 months. Our results, however, could not confirm previous findings on elevated transition risks in CHR-P individuals with combined UHR and BS. However, CHR-P individuals who met both CAARMS and SPI-A criteria at baseline were over 4 times more likely to experience PFO at 12-month follow-up than those who met SPI-A only criteria at baseline. Together, these findings highlight the possible importance of extending the detection of CHR-P individuals outside established clinical pathways as one way to improve the early detection and prevention in psychosis.

## Supplementary Material

sgae029_suppl_Supplementary_Tables_S1-S13_Figures_S1-S2

## Data Availability

The datasets generated and/or analyzed during the current study are available from the corresponding author on reasonable request.
