# Supplementary material for: Clinical and Functional Outcomes of Community-Recruited Individuals at Clinical High-Risk for Psychosis: Results From the Youth Mental Health Risk and Resilience Study (YouR-Study)
Source: Schizophr Bull Open. 2024 Nov 12;5(1):sgae029. doi: 10.1093/schizbullopen/sgae029 (PMC11604080; doi:10.1093/schizbullopen/sgae029)
Supplement: sgae029_suppl_Supplementary_Tables_S1-S13_Figures_S1-S2 [file sgae029_suppl_supplementary_tables_s1-s13_figures_s1-s2.docx]

**Supplementary Material to**

**Clinical and functional outcomes of community-recruited individuals at clinical high-risk for psychosis: Results from the Youth Mental Health Risk and Resilience Study (YouR-Study)**

**by K. Haining et al.**

Table of Contents

[Supplementary Methods 3](#_Toc180568515)

[CAARMS and SPI-A severity/distress 3](#_Toc180568516)

[Cognitive scores 3](#_Toc180568517)

[Current MINI comorbidity categories 3](#_Toc180568518)

[Questionnaire scores 4](#_Toc180568519)

[References 5](#_Toc180568520)

[Supplementary Results 7](#_Toc180568521)

[Diagnostic categories 7](#_Toc180568522)

[CHR-P 7](#_Toc180568523)

[CHR-P transitions 7](#_Toc180568524)

[Longitudinal analyses within the CHR-N group 7](#_Toc180568525)

[Supplementary Table 1. Baseline assessment schedule for study groups 8](#_Toc180568526)

[Supplementary Table 2. Additional clinical, functional and questionnaire data for CHR-P (N = 138), CHR-N (N = 47) and HC (N = 58) participants at baseline 9](#_Toc180568527)

[Supplementary Table 3. Demographic, clinical and functional characteristics of CHR-P individuals with (N = 116) and without (N = 28) follow-up data at baseline 10](#_Toc180568528)

[Supplementary Table 4. Additional clinical, functional and questionnaire data for CHR-P individuals with (N = 116) and without follow-up data (N = 22) at baseline 11](#_Toc180568529)

[Supplementary Table 5. Subgroup analysis of secondary variables over follow-up among CHR-P individuals (N = 64) 12](#_Toc180568530)

[Supplementary Table 6. Subgroup analysis of primary variables over follow-up among CHR-NT individuals (N = 77) 13](#_Toc180568531)

[Supplementary Table 7. Subgroup analysis of secondary variables over follow-up among CHR-NT individuals (N = 59) 14](#_Toc180568532)

[Supplementary Table 8. Subgroup analysis of primary variables over follow-up among CHR-N individuals (N = 20) 15](#_Toc180568533)

[Supplementary Table 9. Subgroup analysis of secondary variables over follow-up among CHR-N individuals (N = 16) 16](#_Toc180568534)

[Supplementary Table 10. Demographic, clinical, functional and cognitive characteristics of CHR-T (N = 14) versus CHR-NT (N = 130) participants at baseline 17](#_Toc180568535)

[Supplementary Table 11. Additional clinical, functional and questionnaire data for CHR-T (N = 14) versus CHR-NT (N = 124) participants at baseline 18](#_Toc180568536)

[Supplementary Table 12. Cumulative hazard rates for transition at each follow-up time point in the different risk criteria subgroups 19](#_Toc180568537)

[Supplementary Table 13. Additional clinical, functional and questionnaire data for PFO (N = 67) versus GFO (N = 49) participants and persistent APS (N = 38) versus non-persistent APS (N = 47) participants at baseline 20](#_Toc180568538)

[Supplementary Figure 1. CHR-P flowchart of baseline and follow-up assessments. 21](#_Toc180568539)

[Supplementary Figure 2. CHR-N flowchart of baseline and follow-up assessments. 22](#_Toc180568540)

# **Supplementary Methods**

## **CAARMS and SPI-A severity/distress**

CAARMS severity was calculated by multiplying the global score by the frequency score for each of the four domains and summing these products while SPI-A severity was calculated by summing the frequency scores for each basic symptom. For SPI-A, frequency scores of 7 and 9 were re-scored to 0 while scores of 8 were re-scored to 1. CAARMS mean distress scores for each participant were calculated by the formula (D_utc_ + D_nbi_ + D_pa_ + D_ds_)/the number of APS reported. SPI-A mean distress scores for each participant were calculated in a similar manner using the formula D_B1_ + D_C2_ + D_C3_ + … + D_O8_)/the number of basic symptoms reported. The maximum CAARMS and SPI-A distress scores were also obtained for each participant.

## **Cognitive scores**

In line with Keefe et al^1^ BACS raw scores for each cognitive domain were converted into standardized z-scores using the means and standard deviations of sex-specific HCs. The BACS composite score was created by averaging the *z*-scores obtained from the six primary measures and then converting this value into a standardised *z*-score as before. For consistency, CNB raw accuracy and RT scores were calculated in the same way, albeit without correction for sex. RT z-scores were multiplied by − 1, to produce speed values where, as for accuracy, higher scores reflect better performance. CNB efficiency scores were then generated for each domain by taking the arithmetic mean of the accuracy and RT z-scores.^2^ Outliers beyond ± 5.0 z-scores were curtailed to values of + 5.0 or − 5.0. In addition, NART-derived estimates of premorbid full-scale IQ were obtained using a recently re-standardised calculation.^3^

## **Current MINI comorbidity categories**

1. Anxiety disorders = panic disorder (past month), agoraphobia (current), social phobia (past month), generalised anxiety disorder (past 6 months)
2. Mood disorders = major depressive episode (past 2 weeks), dysthymia (past 2 year), (hypo)manic episode (current)
3. Alcohol dependence/abuse = alcohol dependence/abuse (past 12 months)
4. Drug dependence/abuse = drug dependence/abuse (past 12 months)
5. Eating disorders = anorexia nervosa (past 3 months), bulimia nervosa (past 3 months)

## **Questionnaire scores**

1. International Positive and Negative Affect Schedule - Short Form (IPANAS-SF)^4^ - Calculated overall total scores per participant i.e., the sum of the scores related to positive affect and the sum of the scores related to negative affect. Higher scores reflect higher levels of positive or negative affect.
2. Inventory of Interpersonal Problems (IIP-32)^5^ - Calculated overall mean scores per participant i.e., the sum of the scores of the 32 items divided by 32. Higher scores reflect greater interpersonal problems.
3. Significant Others Scale (SOS)^6^ - For partner (if applicable), close relative and close friend, we calculated a mean rating for actual practical support, actual emotional support, ideal practical support and ideal emotional support. Discrepancy scores (i.e., ideal support minus actual support) were calculated for practical and emotional support items, collapsed across the three roles (two if no partner), with higher scores indicating greater dissatisfaction with support. Two scores were derived from the discrepancies between actual and ideal perceived support: satisfaction with practical support and satisfaction with emotional support. Negative discrepancy scores, whereby actual support > ideal support, were recoded to zero to account for overprovision of support.
4. Social Interaction Anxiety Scale (SIAS)^7^ - Calculated overall total scores per participant i.e., the sum of the scores of the 20 items. Note that three items were reverse scored. Higher scores reflect greater levels of social interaction anxiety.
5. Adverse Childhood Experiences (ACEs) Questionnaire^8^ – Calculated the total numbers of ACEs experienced in the first 18 years of life per participant, out of a possible total of 10. ACEs comprised abuse (emotional, physical and sexual), neglect (emotional and physical) and various examples of household dysfunction (i.e., household substance abuse, household mental illness, mother treated violently, parental separation or divorce, incarcerated household member).
6. Beliefs About Paranoia Scale (BAPS)^9^ - Calculated overall total scores per participant i.e., the sum of the scores related to negative beliefs, survival beliefs and normalising beliefs. Higher scores reflect greater negative beliefs about paranoia, beliefs about paranoia as a survival strategy and normalising beliefs.
7. Brief Core Schema Scale (BCSS)^10^ – Calculated overall total scores per participant i.e., the sum of the scores related to negative-self, positive-self, negative-other and positive-other. Higher scores reflect greater positive or negative evaluations of self and others.
8. Psychosis Attachment Measure (PAM)^11^ **-** Calculated overall mean scores per participant i.e., the mean of the scores related to anxious attachment and the mean of the scores related to avoidant attachment. Note that three avoidance items were reverse scored. Higher scores reflect greater levels of anxious or avoidant attachment.
9. Rust Inventory of Schizotypal Cognitions (RISC)^12^ - Calculated overall total scores per participant i.e., the sum of the scores of the 26 items. Note that 13 items were reverse scored. Higher scores reflect a higher incidence of schizotypal cognitions.

## **References**

1. Keefe RSE, Harvey PD, Goldberg TE, et al. Norms and standardization of the Brief Assessment of Cognition in Schizophrenia (BACS). *Schizophr Res*. 2008;102(1-3):108-115. doi:10.1016/j.schres.2008.03.024

2. Moore TM, Reise SP, Gur RE, Hakonarson H, Gur RC. Psychometric properties of the Penn Computerized Neurocognitive Battery. *Neuropsychology*. 2015;29(2):235-246. doi:10.1037/neu0000093

3. Bright P, Hale E, Gooch VJ, Myhill T, van der Linde I. The National Adult Reading Test: restandardisation against the Wechsler Adult Intelligence Scale-Fourth edition. *Neuropsychol Rehabil*. 2018;28(6):1019-1027. doi:10.1080/09602011.2016.1231121

4. Thompson ER. Development and validation of an internationally reliable short-form of the Positive and Negative Affect Schedule (PANAS). *J Cross Cult Psychol*. 2007;38(2):227-242. doi:10.1177/0022022106297301

5. Horowitz LM, Alden LE, Wiggins JS, Pincus AL. *Manual for the Inventory of Interpersonal Problems*. Psychological Corporation; 2000.

6. Power MJ, Champion LA, Aris SJ. The development of a measure of social support: The Significant Others (SOS) Scale. *British Journal of Clinical Psychology*. 1988;27(4):349-358. doi:10.1111/j.2044-8260.1988.tb00799.x

7. Mattick RP, Clarke JC. Development and validation of measures of social phobia scrutiny fear and social interaction anxiety. *Behaviour research and therapy*. 1998;36(4):455-470. doi:10.1016/s0005-7967(97)10031-6

8. Murphy A, Steele M, Dube SR, et al. Adverse Childhood Experiences (ACEs) questionnaire and Adult Attachment Interview (AAI): implications for parent child relationships. *Child Abuse Negl*. 2014;38(2):224-233. doi:10.1016/j.chiabu.2013.09.004

9. Gumley AI, Gillan K, Morrison AP, Schwannauer M. The development and validation of the beliefs about paranoia scale (short form). *Behavioural and Cognitive Psychotherapy*. 2011;39(1):35-53. doi:10.1017/S135246581000055X

10. Fowler D, Freeman D, Smith B, et al. The Brief Core Schema Scales (BCSS): Psychometric properties and associations with paranoia and grandiosity in non-clinical and psychosis samples. *Psychol Med*. 2006;36(6):749-759. doi:10.1017/S0033291706007355

11. Berry K, Wearden A, Barrowclough C, Liversidge T. Attachment styles, interpersonal relationships and psychotic phenomena in a non-clinical student sample. *Pers Individ Dif*. 2006;41(4):707-718. doi:10.1016/j.paid.2006.03.009

12. Rust J. The Rust Inventory of Schizotypal Cognitions (RISC). *Schizophr Bull*. 1988;14(2):317-322. doi:10.1093/schbul/14.2.317

# **Supplementary Results**

## **Diagnostic categories**

### ***CHR-P***

At baseline, of those meeting CAARMS, 102 (94.4%) met APS criteria, 3 (2.8%) met GRFD criteria and 3 (2.8%) met both APS and GRFD criteria; while, of those meeting SPI-A criteria, 50 (47.6%) met COPER criteria, 15 (14.3%) met COGDIS criteria and 40 (38.1%) met both. Notably, 34 of 144 (23.6%) CHR-P participants met both APS and COGDIS criteria at baseline.

### ***CHR-P transitions***

The 14 CHR-P individuals who transitioned to psychosis met SCID DSM-IV diagnostic criteria for schizophrenia (n = 3), schizoaffective disorder (n = 4), delusional disorder (n = 2), psychotic disorder not otherwise specified (n = 3), bipolar II with psychotic features (n = 1) and depression with psychotic features (n = 1). At baseline, 64.3% of transitioned cases met both CAARMS/SPI-A criteria, 28.6% met CAARMS criteria only and 7.1% met SPI-A criteria only. In addition, 42.9% of transitioned cases met both APS and COGDIS criteria at baseline.

## **Longitudinal analyses within the CHR-N group**

The longitudinal analyses of primary and secondary variables were repeated for CHR-N participants who provided data at baseline, 6-12 months follow-up and 18-24 months follow-up (N = 20 for primary variables & N = 16 for secondary variables; Supplementary Tables 8 & 9). In the CHR-N group, we found a significant increase in SPI-A severity between baseline and 24 months follow-up and a significant decrease in the highest social functioning scores achieved over the past year between baseline and 24 months follow-up.

# Supplementary Table 1. Baseline assessment schedule for study groups

|  | **CHR-P & CHR-N** | **HC** |
| --- | --- | --- |
| **Visit 1** | Demographics | Demographics |
|  | CAARMS positive scale | CAARMS positive scale |
|  | SPI-A (COGDIS/COPER) | SPI-A (COGDIS/COPER) |
|  | GAF | GAF |
|  |  | MINI |
|  |  | PAS |
|  |  | GF: Social |
|  |  | GF: Role |
| **Visit 2** | MINI | BACS |
|  | PAS | CNB |
|  | GF: Social | NART |
|  | GF: Role | BAPS |
|  |  | BCSS |
|  |  | PAM |
|  |  | ACEs |
|  |  | RISC |
|  |  | IIP-32 |
|  |  | SOS |
|  |  | IPANAS-SF |
|  |  | SIAS |
| **Visit 3** | BACS | Neuroimaging |
|  | CNB |  |
|  | NART |  |
|  | BAPS |  |
|  | BCSS |  |
|  | PAM |  |
|  | ACEs |  |
|  | RISC |  |
| **Visit 4** | IIP-32 |  |
|  | SOS |  |
|  | IPANAS-SF |  |
|  | SIAS |  |
|  | Neuroimaging |  |

ACEs, Adverse Childhood Experiences; BACS, Brief Assessment of Cognition in Schizophrenia; BAPS, Beliefs About Paranoia Scale; BCSS, Brief Core Schema Scale; CAARMS, Comprehensive Assessment of At-Risk Mental States; CHR-N, clinical high-risk-negative; CHR-P, clinical high-risk for psychosis; CNB, Penn Computerized Neurocognitive Battery; COGDIS, Cognitive Disturbances; COPER, Cognitive-Perceptive Basic Symptoms; FEP, first-episode psychosis; GAF, Global Assessment of Functioning; GF, Global Functioning; HC, healthy control; IIP-32, Inventory of Interpersonal Problems; IPANAS-SF, International Positive and Negative Affect Schedule - Short Form; MINI, Mini-International Neuropsychiatric Interview; NART, National Adult Reading Test; PAM, Psychosis Attachment Measure; PAS, Premorbid Adjustment Scale; RISC, Rust Inventory of Schizotypal Cognitions; SD, standard deviation; SIAS, Social Interaction Anxiety Scale; SOS, Significant Others Scale; SPI-A, Schizophrenia Proneness Instrument, Adult version

# Supplementary Table 2. Additional clinical, functional and questionnaire data for CHR-P (N = 138), CHR-N (N = 47) and HC (N = 58) participants at baseline

|  | **CHR-P (1)**  **(N = 138)** | **CHR-N (2)**  **(N = 47)** | **HC (3)**  **(N = 58)** | ***p*** | **Effect size^c^** | **Post hoc**  **Test** |
| --- | --- | --- | --- | --- | --- | --- |
| MINI data, n (%) | | | | | | |
| Major depressive episode (past 2 weeks) | 58 (42.0) | 11 (23.4) | 0 (0) | < .001 | *V* = .386 | 1, 2 > 3 |
| Dysthymia (past 2 year) | 20 (14.5) | 1 (2.1) | 0 (0) | < .001 | *V* = .240 | 1 > 3 |
| Suicide risk (past month) |  |  |  |  |  |  |
| Low | 31 (22.5) | 3 (6.4) | 0 (0) | < .001 | *V* = .286 | 1 > 2, 3 |
| Medium | 21 (15.2) | 3 (6.4) | 0 (0) | .003 | *V* = .217 | 1 > 3 |
| High | 25 (18.1) | 5 (10.6) | 0 (0) | .002 | *V* = .227 | 1, 2 > 3 |
| (Hypo) manic episode (current) | 18 (13.0) | 0 (0) | 0 (0) | < .001 | *V* = .247 | 1 > 2, 3 |
| Panic disorder (past month) | 12 (8.7) | 3 (6.4) | 0 (0) | .049 | *V* = .148 | - |
| Agoraphobia (current) | 54 (39.1) | 4 (8.5) | 0 (0) | < .001 | *V* = .416 | 1 > 2, 3 |
| Social phobia (past month) | 44 (31.9) | 7 (14.9) | 0 (0) | < .001 | *V* = .329 | 1, 2 > 3 |
| Obsessive-compulsive disorder (past month) | 20 (14.5) | 0 (0) | 0 (0) | < .001 | *V* = .261 | 1 > 2, 3 |
| Posttraumatic stress disorder (past month) | 13 (9.4) | 1 (2.1) | 0 (0) | .011 | *V* = .182 | 1 > 3 |
| Alcohol dependence/abuse (past 12 months) | 43 (31.2) | 11 (23.4) | 0 (0) | < .001 | *V* = .308 | 1, 2 > 3 |
| Drug dependence/abuse (past 12 months) | 22 (15.9) | 3 (6.4) | 0 (0) | .002 | *V* = .224 | 1 > 3 |
| Anorexia nervosa (past 3 months) | 2 (1.4) | 0 (0) | 0 (0) | 1.000 | *V* = .079 | - |
| Bulimia nervosa (past 3 months) | 11 (8.0) | 1 (2.1) | 0 (0) | .034 | *V* = .164 | - |
| Generalised anxiety disorder (past 6 months) | 68 (49.3) | 17 (36.2) | 0 (0) | < .001 | *V* = .424 | 1, 2 > 3 |
| Functioning data, median (range) | | | | | | |
| GF: Social lowest | 7 (2-9) | 8 (4-9) | 8.5 (7-9) | < .001 | η^2^*_p_* = .286 | 1 < 2 < 3 |
| GF: Social highest | 8 (4-10) | 9 (6-10) | 9 (8-10) | < .001 | η^2^*_p_* = .180 | 1 < 2 < 3 |
| GF: Role lowest | 7 (3-9) | 8 (4-9) | 9 (5-9) | < .001 | η^2^*_p_* = .226 | 1 < 2 < 3 |
| GF: Role highest | 8 (3-10) | 8 (6-10) | 9 (7-10) | < .001 | η^2^*_p_* = .138 | 1 < 2 < 3 |
| Questionnaire data, mean (SD) | | | | | | |
| Positive affect (IPANAS-SF)^a^ | 25.68 (7.23) | 27.61 (7.26) | 32.58 (7.18) | < .001 | ω² = .129 | 1, 2 < 3 |
| Negative affect (IPANAS-SF)^a^ | 26.18 (7.92) | 22.47 (7.59) | 17.31 (5.71) | < .001 | η^2^*_p_* = .183 | 1 > 2 > 3 |
| Interpersonal problems (IIP-32)^a^ | 1.56 (0.51) | 1.17 (0.51) | 0.81 (0.47) | < .001 | η^2^*_p_* = .231 | 1 > 2 > 3 |
| Practical support satisfaction (SOS)^a^ | 0.97 (0.73) | 0.61 (0.63) | 0.41 (0.37) | < .001 | η^2^*_p_* = .114 | 1 > 2, 3 |
| Emotional support satisfaction (SOS)^a^ | 1.05 (0.77) | 0.73 (0.63) | 1.07 (0.45) | .005 | η^2^*_p_* = .047 | 2 < 1, 3 |
| Social interaction anxiety (SIAS)^a^ | 39.82 (14.00) | 30.22 (14.84) | 20.03 (11.23) | < .001 | η^2^*_p_* = .220 | 1 > 2 > 3 |
| Adverse Childhood Experiences (ACEs)^b^ | 2.17 (2.02) | 1.51 (1.43) | 0.76 (1.05) | < .001 | η^2^*_p_* = .096 | 1, 2 > 3 |
| Negative beliefs (BAPS)^b^ | 13.54 (4.34) | 10.44 (4.69) | 7.89 (2.55) | < .001 | η^2^*_p_* = .226 | 1 > 2 > 3 |
| Survival beliefs (BAPS)^b^ | 11.42 (4.36) | 8.86 (2.59) | 7.84 (2.65) | < .001 | η^2^*_p_* = .144 | 1 > 2, 3 |
| Normalising beliefs (BAPS)^b^ | 16.18 (4.41) | 14.14 (4.38) | 13.16 (4.64) | < .001 | η^2^*_p_* = .072 | 1 > 2, 3 |
| Negative-self (BCSS)^b^ | 7.44 (5.50) | 4.69 (5.56) | 1.38 (2.06) | < .001 | η^2^*_p_* = .215 | 1 > 2 > 3 |
| Positive-self (BCSS)^b^ | 8.24 (4.88) | 11.49 (4.68) | 13.78 (4.78) | < .001 | η^2^*_p_* = .162 | 1 < 2, 3 |
| Negative-other (BCSS)^b^ | 5.98 (5.19) | 3.40 (3.60) | 2.29 (4.64) | < .001 | η^2^*_p_* = .115 | 1 > 2, 3 |
| Positive-other (BCSS)^b^ | 8.61 (4.75) | 9.94 (4.72) | 13.85 (4.84) | < .001 | η^2^*_p_* = .137 | 1, 2 < 3 |
| Attachment anxiety (PAM)^b^ | 1.67 (0.67) | 1.23 (0.62) | 0.85 (0.51) | < .001 | η^2^*_p_* = .187 | 1 > 2 > 3 |
| Attachment avoidance (PAM)^b^ | 1.84 (0.53) | 1.59 (0.61) | 1.17 (0.61) | < .001 | ω² = .181 | 1 > 2 > 3 |
| Schizotypal cognitions (RISC)^b^ | 38.27 (9.44) | 30.32 (10.11) | 22.45 (9.70) | < .001 | ω² = .313 | 1 > 2 > 3 |

ACEs, Adverse Childhood Experiences; BAPS, Beliefs About Paranoia Scale; BCSS, Brief Core Schema Scale; CHR-N, clinical high-risk-negative; CHR-P, clinical high-risk for psychosis; GF, Global Functioning; HC, healthy control; IIP-32, Inventory of Interpersonal Problems; IPANAS-SF, International Positive and Negative Affect Schedule -Short Form; MINI, Mini-International Neuropsychiatric Interview; PAM, Psychosis Attachment Measure; RISC, Rust Inventory of Schizotypal Cognitions; SD, standard deviation; SIAS, Social Interaction Anxiety Scale; SOS, Significant Others Scale

^a^ Refers to n = 124 CHR-P participants and n = 42 CHR-N participants and n = 55 HC participants

^b^ Refers to n = 135 CHR-P participants and n = 47 CHR-N participants and n = 55 HC participants

^c^ Effect sizes were eta squared (η^2^*_p_*) for Kruskal–Wallis *H* tests (small effect = .01, medium effect = .06, large effect = .14), omega squared (ω²) for one-way ANOVAs (small effect = .01, medium effect = .06, large effect = .14) and Cramer's *V* for Pearson’s chi-squared or Fisher–Freeman–Halton exact tests (small effect = .1, medium effect = .3, large effect = .5)

# Supplementary Table 3. Demographic, clinical and functional characteristics of CHR-P individuals with (N = 116) and without (N = 28) follow-up data at baseline

|  | **No follow-ups**  **(N = 28)** | **At least 1 follow-up**  **(N = 116)** | ***p*** | **Effect size**^e^ |
| --- | --- | --- | --- | --- |
| Demographic, clinical and functional data | | | | |
| Age (years), mean (SD) | 20.89 (3.66) | 21.80 (4.25) | .399 | *r* = .070 |
| Sex, female n (%) | 22 (78.6) | 82 (70.7) | .403 | *ϕ* = .070 |
| Education (years), mean (SD) | 14.91 (2.16) | 15.53 (3.07) | .604 | *r* = .043 |
| Current medication, n (%) |  |  |  |  |
| Antidepressant use | 7 (25.0) | 28 (24.1) | .924 | *ϕ* = .008 |
| Anxiolytic use | 3 (10.7) | 12 (10.6) | 1.000 | *ϕ* = .005 |
| Antipsychotic use | 2 (7.1) | 1 (0.9) | .097 | *ϕ* = .174 |
| Current MINI comorbidity, n (%) |  |  |  |  |
| Anxiety disorder | 17 (77.3)^a^ | 86 (74.1) | .757 | *ϕ* = .026 |
| Mood disorder | 16 (72.7)^a^ | 62 (53.4) | .094 | *ϕ* = .142 |
| Alcohol abuse/dependence | 9 (40.9)^a^ | 34 (29.3) | .282 | *ϕ* = .092 |
| Drug abuse/dependence | 5 (22.7)^a^ | 17 (14.7) | .348 | *ϕ* = .081 |
| Eating disorder | 4 (18.2)^a^ | 9 (7.8) | .223 | *ϕ* = .131 |
| Current psychological therapy, n (%) | 6 (21.4) | 20 (17.2) | .605 | *ϕ* = .043 |
| CHR-P criteria met, n (%) |  |  |  |  |
| CAARMS only | 8 (34.8) | 31 (26.7) | .843 | *ϕ* = .016 |
| SPI-A only | 8 (28.6) | 28 (24.1) | .627 | *ϕ* = .041 |
| CAARMS & SPI-A | 12 (42.9) | 57 (49.1) | .550 | *ϕ* = .050 |
| CAARMS severity, median (range) | 28.5 (0-52) | 28 (0-74) | .610 | *r* = .043 |
| CAARMS mean distress, median (range)^b^ | 52.5 (0-85) | 51.3 (0-100) | .729 | *d* = .070 |
| CAARMS max distress, median (range)^b^ | 80 (0-100) | 80 (0-100) | .836 | *r* = .018 |
| SPI-A severity, median (range) | 4.5 (0-33) | 7.5 (0-74) | .069 | *r* = .151 |
| SPI-A mean distress, median (range)^c^ | 33 (0-58) | 32.5 (0-93) | .550 | *r* = .053 |
| SPI-A max distress, median (range)^c^ | 50 (0-90) | 50 (0-100) | .395 | *r* = .076 |
| GAF, median (range) | 58 (38-95) | 58.5 (21-91) | .396 | *r* = .071 |
| PAS, median (range) | 1.04 (0-3)^a^ | 1.21 (0-3) | .767 | *r* = .025 |
| GF: Social current, median (range) | 8 (5-9)^a^ | 8 (3-10) | .825 | *r* = .019 |
| GF: Role current, median (range) | 8 (4-9)^a^ | 8 (3-9) | .384 | *r* = .074 |
| Cognitive data, mean (SD) | | | | |
| Premorbid IQ | 106.79 (8.25)^d^ | 110.68 (6.73) | .064 | *d* = 0.560 |
| Verbal memory | -0.29 (1.32)^d^ | -0.05 (1.14) | .465 | *d* = 0.205 |
| Working memory | 0.13 (1.16)^d^ | -0.03 (1.36) | .773 | *r* = .025 |
| Motor speed | -0.22 (0.91)^d^ | -0.68 (1.10) | .057 | *d* = 0.429 |
| Verbal fluency | -0.18 (1.11)^d^ | -0.09 (0.90) | .752 | *r* = .027 |
| Attention and processing speed | -0.49 (1.16)^d^ | -0.38 (1.11) | .711 | *r* = .032 |
| Executive function | 0.21 (1.63)^d^ | 0.01 (1.10) | .315 | *r* = .086 |
| BACS total | -0.32 (1.64)^d^ | -0.46 (1.53) | .745 | *d* = 0.086 |
| Emotion recognition efficiency | -0.39 (0.81)^d^ | -0.29 (0.93) | .440 | *r* = .066 |
| Working memory efficiency | 0.01 (0.75)^d^ | -0.09 (0.83) | .745 | *r* = .028 |
| Attention efficiency | 0.08 (0.90)^d^ | -0.10 (0.88) | .194 | *r* = .112 |

APS, attenuated psychotic symptoms; BACS, Brief Assessment of Cognition in Schizophrenia; CAARMS, Comprehensive Assessment of At-Risk Mental States; CHR-P, clinical high-risk for psychosis; GAF, Global Assessment of Functioning; GF, Global Functioning; MINI, Mini-International Neuropsychiatric Interview; PAS, Premorbid Adjustment Scale; SCID, Structured Clinical Interview for DSM-IV; SPI-A, Schizophrenia Proneness Instrument, Adult version

^a^ Refers to n = 22 CHR-P participants i.e., those with Visit 2 data

^b^ Refers to n = 139 CHR-P participants (with follow-up = 112, without follow-up = 27) i.e., those with CAARMS severity > 0

^c^ Refers to n = 127 CHR-P participants (with follow-up = 104, without follow-up = 23) i.e., those with SPI-A severity > 0

^d^ Refers to n = 19 CHR-P participants i.e., those with cognitive data

^e^ Effect sizes were Rosenthal's *r* for Mann-Whitney U tests (small effect = .1, medium effect = .3, large effect = .5), Cohen’s *d* for Welch’s *t*-tests (small effect = 0.2, medium effect = 0.5, large effect = 0.8) and Phi (*ϕ*) for Pearson’s chi-squared or Fisher′s exact tests (small effect = .1, medium effect = .3, large effect = .5)

# Supplementary Table 4. Additional clinical, functional and questionnaire data for CHR-P individuals with (N = 116) and without follow-up data (N = 22) at baseline

|  | **No follow-ups**  **(N = 22)** | **At least 1 follow-up**  **(N = 116)** | ***p*** | **Effect size**^c^ |
| --- | --- | --- | --- | --- |
| MINI data, n (%) | | | | |
| Major depressive episode (past 2 weeks) | 13 (59.1) | 45 (38.8) | .077 | *ϕ* = .151 |
| Dysthymia (past 2 year) | 2 (9.1) | 18 (15.5) | .741 | *ϕ* = .067 |
| Suicide risk (past month) |  |  |  |  |
| Low | 7 (31.8) | 24 (20.7) | .271 | *ϕ* = .098 |
| Medium | 2 (9.1) | 19 (16.4) | .527 | *ϕ* = .074 |
| High | 4 (18.2) | 21 (18.1) | 1.000 | *ϕ* = .001 |
| (Hypo) manic episode (current) | 3 (13.6) | 15 (12.9) | 1.000 | *ϕ* = .008 |
| Panic disorder (past month) | 4 (18.2) | 8 (6.9) | .101 | *ϕ* = .147 |
| Agoraphobia (current) | 10 (45.5) | 44 (37.9) | .507 | *ϕ* = .056 |
| Social phobia (past month) | 10 (45.5) | 34 (29.3) | .136 | *ϕ* = .127 |
| Obsessive-compulsive disorder (past month) | 2 (9.1) | 18 (15.5) | .741 | *ϕ* = .067 |
| Posttraumatic stress disorder (past month) | 3 (13.6) | 10 (8.6) | .436 | *ϕ* = .063 |
| Alcohol dependence/abuse (past 12 months) | 9 (40.9) | 34 (29.3) | .282 | *ϕ* = .092 |
| Drug dependence/abuse (past 12 months) | 5 (22.7) | 17 (14.7) | .348 | *ϕ* = .081 |
| Anorexia nervosa (past 3 months) | 1 (4.5) | 1 (0.86) | .294 | *ϕ* = .113 |
| Bulimia nervosa (past 3 months) | 3 (13.6) | 8 (6.9) | .382 | *ϕ* = .091 |
| Generalised anxiety disorder (past 6 months) | 12 (54.5) | 56 (48.3) | .590 | *ϕ* = .046 |
| Functioning data, median (range) | | | | |
| GF: Social lowest | 6.5 (2-9) | 7 (3-9) | .381 | *r* = .075 |
| GF: Social highest | 8 (7-10) | 8 (4-10) | .731 | *r* = 029 |
| GF: Role lowest | 7 (4-9) | 7 (3-9) | .325 | *r* = .084 |
| GF: Role highest | 8 (5-9) | 8 (3-10) | .555 | *r* = .050 |
| Questionnaire data, mean (SD) | | | | |
| Positive affect (IPANAS-SF)^a^ | 27.96 (9.44) | 25.48 (7.02) | .438 | *d* = 0.342 |
| Negative affect (IPANAS-SF)^a^ | 26.00 (7.01) | 26.20 (8.02) | .934 | *d* = 0.025 |
| Interpersonal problems (IIP-32)^a^ | 1.68 (0.36) | 1.55 (0.52) | .332 | *d* = 0.248 |
| Practical support satisfaction (SOS)^a^ | 1.04 (0.73) | 0.96 (0.73) | .597 | *r* = .048 |
| Emotional support satisfaction (SOS)^a^ | 0.91 (0.54) | 1.06 (0.79) | .804 | *r* = .022 |
| Social interaction anxiety (SIAS)^a^ | 42.70 (11.08) | 39.56 (14.24) | .535 | *r* = .056 |
| Adverse Childhood Experiences (ACEs) | 2.68 (2.36)^b^ | 2.09 (1.95) | .333 | *r* = .083 |
| Negative beliefs (BAPS) | 13.63 (4.50)^b^ | 13.52 (4.34) | .977 | *r* = .008 |
| Survival beliefs (BAPS) | 11.53 (4.31)^b^ | 11.40 (4.39) | .814 | *r* = .020 |
| Normalising beliefs (BAPS) | 14.79 (4.30)^b^ | 16.41 (4.40) | .096 | *r* = .143 |
| Negative-self (BCSS) | 7.42 (4.46)^b^ | 7.45 (5.67) | .783 | *r* = .024 |
| Positive-self (BCSS) | 7.74 (4.20)^b^ | 8.33 (4.99) | .673 | *r* = .036 |
| Negative-other (BCSS) | 7.11 (4.64)^b^ | 5.79 (5.28) | .209 | *r* = .108 |
| Positive-other (BCSS) | 9.26 (4.78)^b^ | 8.50 (4.76) | .547 | *r* = .052 |
| Attachment anxiety (PAM) | 1.84 (0.71)^b^ | 1.64 (0.67) | .320 | *r* = .086 |
| Attachment avoidance (PAM) | 1.86 (0.52)^b^ | 1.83 (0.53) | .816 | *d* = 0.057 |
| Schizotypal cognitions (RISC) | 42.53 (7.31)^b^ | 37.58 (9.59) | .014 | *d* = 0.532 |

ACEs, Adverse Childhood Experiences; BAPS, Beliefs About Paranoia Scale; BCSS, Brief Core Schema Scale; CHR-P, clinical high-risk for psychosis; GF, Global Functioning; IIP-32, Inventory of Interpersonal Problems; IPANAS-SF, International Positive and Negative Affect Schedule - Short Form; MINI, Mini-International Neuropsychiatric Interview; PAM, Psychosis Attachment Measure; RISC, Rust Inventory of Schizotypal Cognitions; SD, standard deviation; SIAS, Social Interaction Anxiety Scale; SOS, Significant Others Scale

^a^ Refers to n = 124 CHR-P participants (with follow-up = 114, without follow-up = 10)

^b^ Refers to n = 19 CHR-P participants

^c^ Effect sizes were Rosenthal's *r* for Mann-Whitney U tests (small effect = .1, medium effect = .3, large effect = .5), Cohen’s *d* for Welch’s *t*-tests (small effect = 0.2, medium effect = 0.5, large effect = 0.8) and Phi (*ϕ*) for Pearson’s chi-squared or Fisher′s exact tests (small effect = .1, medium effect = .3, large effect = .5)

# Supplementary Table 5. Subgroup analysis of secondary variables over follow-up among CHR-P individuals (N = 64)

|  | **Baseline**  **(1)** | **6-12 months**  **(2)** | **24 months**  **(3)** | ***p*** | **Effect size**^c^ |
| --- | --- | --- | --- | --- | --- |
| SPI-A severity, median (range)^a^ | 7 (0-39) | - | 6 (0-22) | .093 |  |
| SPI-A mean distress, median (range)^b^ | 36.7 (0-93) | - | 30 (0-95) | .633 |  |
| SPI-A max distress, median (range)^b^ | 60 (0-100) | - | 50 (0-100) | .400 |  |
| BS criteria met, n (%)^a^ |  |  |  |  |  |
| COGDIS only | 5 (10.9) | - | 2 (4.3) | .375 |  |
| COPER only | 20 (43.5) | - | 9 (19.6) | .013 | *g* = 0.324 |
| COGDIS & COPER | 11 (23.9) | - | 14 (30.4) | .581 |  |
| BS persistence^a^ | - | - | 24 (52.2) | - |  |
| GF: Social current, median (range) | 8 (3-9) | 8 (5-10) | 8 (3-9) | .412 |  |
| GF: Social lowest, median (range) | 7 (3-9) | 7 (3-9) | 7 (3-9) | .129 |  |
| GF: Social highest, median (range) | 8 (5-10) | 8 (6-10) | 8 (6-9) | .063 |  |
| GF: Role current, median (range) | 8 (3-9) | 8 (4-9) | 8 (5-10) | .349 |  |
| GF: Role lowest, median (range) | 7 (3-9) | 7 (4-9) | 7.5 (4-9) | .310 |  |
| GF: Role highest, median (range) | 8 (3-10) | 8 (5-10) | 8 (6-10) | .377 |  |
| MINI current MDE, n (%) | 24 (37.5) | - | - | - |  |
| SCID current MDE, n (%) | - | 10 (15.6) | 12 (18.8) | .791 |  |
| MDE persistence, n (%) | - | 6 (9.4) | 7 (10.9) | 1.000 |  |

BS, basic symptoms; CHR-P, clinical high-risk for psychosis; COGDIS, Cognitive Disturbances; COPER, Cognitive-Perceptive Basic Symptoms; GF, Global Functioning; MDE; major depressive episode; MINI, Mini-International Neuropsychiatric Interview; SCID, Structured Clinical Interview for DSM-IV; SPI-A, Schizophrenia Proneness Instrument, Adult version

^a^ Refers to n = 46 CHR-P participants who had SPI-A data at baseline and 24 months

^b^ Refers to n = 33 CHR-P participants i.e., those with a SPI-A severity score > 0 across all timepoints

^c^ Effect sizes were Rosenthal's r for Wilcoxon signed rank tests (small effect = .1, medium effect = .3, large effect = .5), Cohen’s *g* for McNemar’s test (small effect = 0.05, medium effect = 0.15, large effect = 0.25) and Cohen’s *d* for paired t-tests (small effect = 0.2, medium effect = 0.5, large effect = 0.8)

# Supplementary Table 6. Subgroup analysis of primary variables over follow-up among CHR-NT individuals (N = 77)

|  | **Baseline** | **6-12 months** | **18-24 months** | ***p*** | **1 vs 2** | | **1 vs 3** | | **2 vs 3** | |
| --- | --- | --- | --- | --- | --- | --- | --- | --- | --- | --- |
|  | **(1)** | **(2)** | **(3)** |  | ***p*** | **ES**^d^ | ***p*** | **ES**^d^ | ***p*** | **ES**^d^ |
| Clinical and functional data | | | | | | | | | | |
| CAARMS severity, median (range) | 28 (0-72) | 9 (0-80) | 10 (0-66) | < .001 | < .001 | *r* = .636 | < .001 | *r* = .703 | .776 | *r* = .032 |
| CAARMS mean distress, median (range)^a^ | 53.5 (0-97.5) | 40 (0-90) | 50 (0-100) | .045 | .003 | *r* = .398 | .071 | *r* =.241 | .267 | *r* = .148 |
| CAARMS max distress, median (range)^a^ | 80 (0-100) | 60.5 (0-100) | 70 (0-100) | .003 | < .001 | *r* = .485 | .004 | *r* = .384 | .418 | *r* = .108 |
| UHR criteria met, n (%)^b^ |  |  |  |  |  |  |  |  |  |  |
| APS only | 54 (70.1) | 30 (39.0) | 28 (36.4) | < .001 | < .001 | *g* = 0.353 | < .001 | *g* = 0.361 | .850 | *g* = 0.036 |
| BLIPS only | 0 (0) | 1 (1.3) | 0 (0) | 1.000 |  |  |  |  |  |  |
| GRFD only | 2 (2.6) | 0 (0) | 0 (0) | .333 |  |  |  |  |  |  |
| APS Persistence, n (%) |  | 25 (32.5) | 23 (29.9) | .832 |  |  |  |  |  |  |
| GAF, median (range) | 60 (21-91) | 61 (21-87) | 60 (21-88) | .540 |  |  |  |  |  |  |
| Questionnaire data^c^ | | | | | | | | | | |
| Positive affect, mean (SD) | 25.97 (7.24) | 26.91 (9.22) | 26.49 (7.83) | .548 |  |  |  |  |  |  |
| Negative affect, mean (SD) | 25.78 (8.65) | 23.67 (6.64) | 24.20 (6.92) | .060 |  |  |  |  |  |  |
| Interpersonal problems, mean (SD) | 1.49 (0.50) | 1.32 (0.57) | 1.31 (0.56) | < .001 | .001 | *d* = 0.386 | .001 | *d* = 0.394 | .961 | *d* = 0.006 |
| Practical support satisfaction, mean (SD) | 0.97 (0.69) | 0.76 (0.61) | 0.78 (0.65) | .035 | .023 | *d* = 0.267 | .043 | *d* = 0.237 | .814 | *d* = 0.027 |
| Emotional support satisfaction, mean (SD) | 1.07 (0.75) | 0.89 (0.66) | 0.98 (0.76) | .123 |  |  |  |  |  |  |

APS, attenuated psychotic symptoms; BLIPS, brief limited intermittent psychotic symptoms; CAARMS, Comprehensive Assessment of At-Risk Mental States; CHR-NT, clinical high-risk non-transitions; GAF, Global Assessment of Functioning; GRFD, genetic risk and functional deterioration; UHR, ultra-high risk

**^a^** Refers to n = 56 CHR-NT participants i.e., those with a CAARMS severity score > 0 across all timepoints

^b^ UHR criteria for APS, BLIPS and GRFD are not mutually exclusive

^c^ Refers to n = 75 CHR-NT participants i.e., individuals with questionnaire data across all timepoints

^d^ Effect sizes were Rosenthal's *r* for Wilcoxon signed rank tests (small effect = .1, medium effect = .3, large effect = .5), Cohen’s *g* for McNemar’s test (small effect = 0.05, medium effect = 0.15, large effect = 0.25) and Cohen’s *d* for paired t-tests (small effect = 0.2, medium effect = 0.5, large effect = 0.8)

# Supplementary Table 7. Subgroup analysis of secondary variables over follow-up among CHR-NT individuals (N = 59)

|  | **Baseline**  **(1)** | **6-12 months**  **(2)** | **24 months**  **(3)** | ***p*** | **Effect size**^c^ |
| --- | --- | --- | --- | --- | --- |
| SPI-A severity, median (range)^a^ | 7 (0-39) | - | 6 (0-22) | .296 |  |
| SPI-A mean distress, median (range)^b^ | 35 (0-93) | - | 30 (0-95) | .869 |  |
| SPI-A max distress, median (range)^b^ | 60 (0-100) | - | 50 (0-100) | .535 |  |
| BS criteria met, n (%)^a^ |  |  |  |  |  |
| COGDIS only | 5 (11.6) | - | 2 (4.7) | .375 |  |
| COPER only | 20 (46.5) | - | 8 (18.6) | .004 | *g* = 0.375 |
| COGDIS & COPER | 8 (18.6) | - | 13 (30.2) | .227 |  |
| BS persistence^a^ | - | - | 22 (51.2) | - |  |
| GF: Social current, median (range) | 8 (3-9) | 8 (5-10) | 8 (3-9) | .283 |  |
| GF: Social lowest, median (range) | 7 (3-9) | 7 (3-9) | 7 (3-9) | .065 |  |
| GF: Social highest, median (range) | 8 (5-10) | 8 (6-10) | 8 (6-9) | .084 |  |
| GF: Role current, median (range) | 8 (3-9) | 8 (4-9) | 8 (5-10) | .426 |  |
| GF: Role lowest, median (range) | 7 (3-9) | 7 (4-9) | 8 (4-9) | .254 |  |
| GF: Role highest, median (range) | 8 (3-10) | 8 (5-10) | 8 (6-10) | .389 |  |
| MINI current MDE, n (%) | 20 (33.9) | - | - | - |  |
| SCID current MDE, n (%) | - | 9 (15.3) | 10 (16.9) | 1.000 |  |
| MDE persistence, n (%) | - | 5 (8.5) | 5 (8.5) | 1.000 |  |

BS, basic symptoms; CHR-NT, clinical high-risk non-transitions; COGDIS, Cognitive Disturbances; COPER, Cognitive-Perceptive Basic Symptoms; GF, Global Functioning; MDE; major depressive episode; MINI, Mini-International Neuropsychiatric Interview; SCID, Structured Clinical Interview for DSM-IV; SPI-A, Schizophrenia Proneness Instrument, Adult version

^a^ Refers to n = 43 CHR-NT participants who had SPI-A data at baseline and 24 months

^b^ Refers to n = 31 CHR-NT participants i.e., those with a SPI-A severity score > 0 across all timepoints

^c^ Effect sizes were Rosenthal's *r* for Wilcoxon signed rank tests (small effect = .1, medium effect = .3, large effect = .5), Cohen’s *g* for McNemar’s test (small effect = 0.05, medium effect = 0.15, large effect = 0.25) and Cohen’s *d* for paired t-tests (small effect = 0.2, medium effect = 0.5, large effect = 0.8)

# Supplementary Table 8. Subgroup analysis of primary variables over follow-up among CHR-N individuals (N = 20)

|  | **Baseline**  **(1)** | **6-12 months**  **(2)** | **18-24 months**  **(3)** | ***p*** | **Effect size**^c^ |
| --- | --- | --- | --- | --- | --- |
| Clinical and functional data | | | | | |
| CAARMS severity, median (range) | 5 (0-24) | 2 (0-49) | 0 (0-36) | .278 |  |
| CAARMS mean distress, median (range)^a^ | 29.8 (0-73.3) | 14.5 (0-70) | 43.6 (0-100) | .738 |  |
| CAARMS max distress, median (range)^a^ | 53 (0-90) | 24 (0-80) | 70 (0-100) | .580 |  |
| UHR criteria met, n (%) |  |  |  |  |  |
| APS only | - | 5 (25.0) | 4 (20.0) | 1.000 |  |
| BLIPS only | - | 0 (0) | 0 (0) | - |  |
| GRFD only | - | 0 (0) | 0 (0) | - |  |
| GAF, median (range) | 66.5 (43-88) | 71.5 (21-89) | 72 (21-90) | .426 |  |
| Questionnaire data^b^ | | | | | |
| Positive affect, mean (SD) | 27.66 (8.54) | 29.16 (7.97) | 24.83 (4.81) | .117 |  |
| Negative affect, mean (SD) | 23.62 (8.92) | 20.42 (8.36) | 21.94 (7.06) | .294 |  |
| Interpersonal problems, mean (SD) | 1.17 (0.57) | 1.09 (0.52) | 1.04 (0.52) | .603 |  |
| Practical support satisfaction, mean (SD) | 0.58 (0.55) | 0.54 (0.60) | 0.74 (0.69) | .272 |  |
| Emotional support satisfaction, mean (SD) | 0.63 (0.50) | 0.67 (0.58) | 0.68 (0.74) | .904 |  |

APS, attenuated psychotic symptoms; BLIPS, brief limited intermittent psychotic symptoms; CAARMS, Comprehensive Assessment of At-Risk Mental States; CHR-N, clinical high-risk-negative; GAF, Global Assessment of Functioning; GRFD, genetic risk and functional deterioration; UHR, ultra-high risk

**^a^** Refers to n = 6 CHR-N participants i.e., those with a CAARMS severity score > 0 across all timepoints

^b^ Refers to n = 18 CHR-N participants i.e., individuals with questionnaire data across all timepoints

^c^ Effect sizes were Rosenthal's *r* for Wilcoxon signed rank tests (small effect = .1, medium effect = .3, large effect = .5), Cohen’s *g* for McNemar’s test (small effect = 0.05, medium effect = 0.15, large effect = 0.25) and Cohen’s *d* for paired t-tests (small effect = 0.2, medium effect = 0.5, large effect = 0.8)

# Supplementary Table 9. Subgroup analysis of secondary variables over follow-up among CHR-N individuals (N = 16)

|  | **Baseline**  **(1)** | **6-12 months**  **(2)** | **24 months**  **(3)** | ***p*** | **Effect size**^c^ |
| --- | --- | --- | --- | --- | --- |
| SPI-A severity, median (range)^a^ | 0 (0-6) | - | 2 (0-9) | .035 | *r* = .585 |
| SPI-A mean distress, median (range)^b^ | 30 (0-70) | - | 43.8 (15-70) | .715 |  |
| SPI-A max distress, median (range)^b^ | 50 (0-70) | - | 50 (30-90) | .465 |  |
| BS criteria met, n (%)^a^ |  |  |  |  |  |
| COGDIS only | - | - | 0 (0) | - |  |
| COPER only | - | - | 3 (18.8) | - |  |
| COGDIS & COPER | - | - | 1 (7.7) | - |  |
| GF: Social current, median (range) | 8 (6-9) | 8 (6-9) | 8 (7-9) | .423 |  |
| GF: Social lowest, median (range) | 8 (4-9) | 7.5 (3-9) | 8 (5-9) | .498 |  |
| GF: Social highest, median (range) | 9 (7-9) | 8 (6-9) | 8 (7-9) | .027 | *r* = .622 |
| GF: Role current, median (range) | 8 (7-9) | 8 (7-9) | 8 (6-9) | .465 |  |
| GF: Role lowest, median (range) | 8 (4-9) | 7 (4-9) | 8 (5-9) | .480 |  |
| GF: Role highest, median (range) | 8 (7-9) | 8 (7-9) | 8 (6-9) | .622 |  |
| MINI current MDE, n (%) | 5 (31.3) | - | - | - |  |
| SCID current MDE, n (%) | - | 0 (0) | 0 (0) | - |  |
| MDE persistence, n (%) | - | 0 (0) | 0 (0) | - |  |

BS, basic symptoms; CHR-N, clinical high-risk-negative; COGDIS, Cognitive Disturbances; COPER, Cognitive-Perceptive Basic Symptoms; GF, Global Functioning; MDE; major depressive episode; MINI, Mini-International Neuropsychiatric Interview; SCID, Structured Clinical Interview for DSM-IV; SPI-A, Schizophrenia Proneness Instrument, Adult version

^a^ Refers to n = 13 CHR-N participants who had SPI-A data at baseline and 24 months

^b^ Refers to n = 4 CHR-N participants i.e., those with a SPI-A severity score > 0 across all timepoints

^c^ Effect sizes were Rosenthal's *r* for Wilcoxon signed rank tests (small effect = .1, medium effect = .3, large effect = .5), Cohen’s *g* for McNemar’s test (small effect = 0.05, medium effect = 0.15, large effect = 0.25) and Cohen’s *d* for paired t-tests (small effect = 0.2, medium effect = 0.5, large effect = 0.8)

# Supplementary Table 10. Demographic, clinical, functional and cognitive characteristics of CHR-T (N = 14) versus CHR-NT (N = 130) participants at baseline

|  | **CHR-T**  **(N = 14)** | **CHR-NT**  **(N = 130)** | ***p*** | **Effect size**^e^ |
| --- | --- | --- | --- | --- |
| Demographic, clinical and functional data | | | | |
| Age (years), mean (SD) | 20.79 (4.46) | 21.72 (4.12) | .287 | *r* = .089 |
| Sex, female n (%) | 11 (78.6) | 93 (71.5) | .758 | *ϕ* = .047 |
| Education (years), mean (SD) | 14.71 (2.37) | 15.49 (2.97) | .379 | *r* = .073 |
| Current medication, n (%) |  |  |  |  |
| Antidepressant use | 3 (21.4) | 32 (24.6) | 1.000 | *ϕ =* .022 |
| Anxiolytic use | 0 (0) | 15 (11.5) | .362 | *ϕ* = .112 |
| Antipsychotic use | 0 (0) | 3 (2.3) | 1.000 | *ϕ =* .048 |
| Current MINI comorbidity, n (%)^a^ |  |  |  |  |
| Anxiety disorder | 13 (92.9) | 90 (72.6) | .117 | *ϕ* = .141 |
| Mood disorder | 13 (92.9) | 65 (52.4) | .004 | *ϕ* = .246 |
| Alcohol abuse/dependence | 9 (64.3) | 34 (27.4) | .011 | *ϕ* = .240 |
| Drug abuse/dependence | 3 (21.4) | 19 (15.3) | .698 | *ϕ* = .050 |
| Eating disorder | 2 (14.3) | 11 (8.9) | .622 | *ϕ* = .056 |
| Current psychological therapy, n (%) | 2 (14.3) | 24 (18.5) | 1.000 | *ϕ* = .032 |
| CHR-P criteria met, n (%) |  |  |  |  |
| CAARMS only | 4 (28.6) | 35 (26.9) | 1.000 | *ϕ* = .011 |
| SPI-A only | 1 (7.1) | 35 (26.9) | .190 | *ϕ* = .135 |
| CAARMS & SPI-A | 9 (64.3) | 60 (46.2) | .197 | *ϕ* = .108 |
| CAARMS severity, median (range) | 42.5 (4-58) | 28 (0-74) | .069 | *r* = .152 |
| CAARMS mean distress, median (range)^b^ | 48.8 (17.5-70) | 52.5 (0-100) | .552 | *r* = .050 |
| CAARMS max distress, median (range) ^b^ | 80 (31-100) | 80 (0-100) | .732 | *r* = .029 |
| SPI-A severity, median (range) | 12 (0-37) | 7 (0-74) | .242 | *r* = .097 |
| SPI-A mean distress, median (range)^c^ | 35 (5-60) | 33 (0-93) | .961 | *r* = .004 |
| SPI-A max distress, median (range)^c^ | 60 (5-99) | 50 (0-100) | .500 | *r* = .057 |
| GAF, median (range) | 51 (41-64) | 58 (21-95) | .037 | *r* = .174 |
| PAS, median (range)^a^ | 1.54 (1-3) | 1.14 (0-3) | .040 | *r* = .175 |
| GF: Social current, median (range)^a^ | 7 (5-8) | 8 (3-10) | .019 | *r* = .200 |
| GF: Role current, median (range)^a^ | 7 (5-9) | 8 (3-9) | .109 | *r* = .136 |
| Cognitive data, mean (SD)^d^ | | | | |
| Premorbid IQ | 110.00 (6.38) | 110.15 (7.16) | .936 | *d* = .021 |
| Verbal memory | -0.34 (0.84) | -0.06 (1.20) | .236 | *r* = .102 |
| Working memory | -0.55 (1.41) | 0.05 (1.31) | .131 | *r* = .130 |
| Motor speed | -0.67 (1.36) | -0.61 (1.05) | .883 | *d* = .052 |
| Verbal fluency | -0.39 (0.55) | -0.06 (0.96) | .176 | *r* = .117 |
| Attention and processing speed | -0.66 (0.58) | -0.37 (1.16) | .129 | *d* = 0.262 |
| Executive function | 0.20 (0.88) | 0.02 (1.21) | .824 | *r* = .019 |
| BACS total | -0.90 (0.97) | -0.38 (1.58) | .094 | *d* = 0.338 |
| Emotion recognition efficiency | -0.67 (1.17) | -0.26 (0.88) | .164 | *r* = .120 |
| Working memory efficiency | -0.18 (0.69) | -0.06 (0.83) | .350 | *r* = .080 |
| Attention efficiency | -0.21 (0.89) | -0.06 (0.89) | .576 | *r* = .048 |

BACS, Brief Assessment of Cognition in Schizophrenia; CAARMS, Comprehensive Assessment of At-Risk Mental States; CHR-NT, clinical high-risk non-transitions; CHR-T, clinical high-risk transitions; GAF, Global Assessment of Functioning; GF, Global Functioning; MINI, Mini-International Neuropsychiatric Interview; PAS, Premorbid Adjustment Scale; SCID, Structured Clinical Interview for DSM-IV; SPI-A, Schizophrenia Proneness Instrument, Adult version

^a^ Refers to n = 138 CHR-P participants (CHR-T = 14, CHR-NT = 124) i.e., those with Visit 2 data

^b^ Refers to n = 139 CHR-P participants (CHR-T = 14, CHR-NT = 125) i.e., those with CAARMS severity > 0

^c^ Refers to n = 127 CHR-P participants (CHR-T = 12, CHR-NT = 115) i.e., those with SPI-A severity > 0

^d^ Refers to n = 135 CHR-P participants (CHR-T = 14, CHR-NT = 121) i.e., those with cognitive data

^e^ Effect sizes were Rosenthal's *r* for Mann-Whitney U tests (small effect = .1, medium effect = .3, large effect = .5), Cohen’s *d* for Welch’s *t*-tests (small effect = 0.2, medium effect = 0.5, large effect = 0.8) and Phi (*ϕ*) for Pearson’s chi-squared or Fisher′s exact tests (small effect = .1, medium effect = .3, large effect = .5)

# Supplementary Table 11. Additional clinical, functional and questionnaire data for CHR-T (N = 14) versus CHR-NT (N = 124) participants at baseline

|  | **CHR-T**  **(N = 14)** | **CHR-NT**  **(N = 124)** | ***P*** | **Effect size**^c^ | |
| --- | --- | --- | --- | --- | --- |
| MINI data, n (%) | | | | |  |
| Major depressive episode (past 2 weeks) | 11 (78.6) | 47 (37.9) | .003 | *ϕ* = .249 | |
| Dysthymia (past 2 year) | 3 (21.4) | 17 (13.7) | .428 | *ϕ* = .066 | |
| Suicide risk (past month) |  |  |  |  | |
| Low | 3 (21.4) | 28 (22.6) | 1.000 | *ϕ* = .008 | |
| Medium | 3 (21.4) | 18 (14.5) | .448 | *ϕ* = .058 | |
| High | 3 (21.4) | 22 (17.7) | .719 | *ϕ* = .029 | |
| (Hypo) manic episode (current) | 3 (21.4) | 15 (12.1) | .395 | *ϕ* = .084 | |
| Panic disorder (past month) | 1 (7.1) | 11 (8.9) | 1.000 | *ϕ* = .019 | |
| Agoraphobia (current) | 5 (35.7) | 49 (39.5) | .782 | *ϕ* = .024 | |
| Social phobia (past month) | 6 (42.9) | 38 (30.6) | .374 | *ϕ* = .079 | |
| Obsessive-compulsive disorder (past month) | 4 (28.6) | 16 (12.9) | .122 | *ϕ* = .134 | |
| Posttraumatic stress disorder (past month) | 1 (7.1) | 12 (9.7) | 1.000 | *ϕ* = .026 | |
| Alcohol dependence/abuse (past 12 months) | 9 (64.3) | 34 (27.4) | .011 | *ϕ* = .240 | |
| Drug dependence/abuse (past 12 months) | 3 (21.4) | 19 (15.3) | .698 | *ϕ* = .050 | |
| Anorexia nervosa (past 3 months) | 0 (0) | 2 (1.6) | 1.000 | *ϕ* = .041 | |
| Bulimia nervosa (past 3 months) | 2 (14.3) | 9 (7.3) | .309 | *ϕ* = .078 | |
| Generalised anxiety disorder (past 6 months) | 9 (64.3) | 59 (47.6) | .236 | *ϕ* = .101 | |
| Functioning data, median (range) | | | | |  |
| GF: Social lowest | 6.5 (4-8) | 7 (2-9) | .114 | *r* = .134 | |
| GF: Social highest | 8 (6-9) | 8 (4-10) | .087 | *r* = .146 | |
| GF: Role lowest | 7 (5-8) | 7 (3-9) | .304 | *r* = .088 | |
| GF: Role highest | 8 (5-9) | 8 (3-10) | .294 | *r* = .089 | |
| Questionnaire data, mean (SD) | | | | |  |
| Positive affect (IPANAS-SF)^a^ | 23.24 (6.44) | 25.99 (7.29) | .156 | *d* = 0.382 | |
| Negative affect (IPANAS-SF)^a^ | 27.24 (6.91) | 25.05 (8.05) | .492 | *r* = 0.062 | |
| Interpersonal problems (IIP-32)^a^ | 1.66 (0.68) | 1.55 (0.48) | .573 | *d* = 0.213 | |
| Practical support satisfaction (SOS)^a^ | 0.71 (0.75) | 1.00 (0.72) | .121 | *r* = .139 | |
| Emotional support satisfaction (SOS)^a^ | 0.85 (0.85) | 1.08 (0.76) | .178 | *r* = .121 | |
| Social interaction anxiety (SIAS)^a^ | 43.13 (16.11) | 39.39 (13.73) | .418 | *r* = .073 | |
| Adverse Childhood Experiences (ACEs)^b^ | 2.00 (1.88) | 2.19 (2.04) | .863 | *r* = .015 | |
| Negative beliefs (BAPS)^b^ | 14.71 (3.71) | 13.40 (4.41) | .193 | *r* = .112 | |
| Survival beliefs (BAPS)^b^ | 10.86 (3.92) | 11.48 (4.42) | .607 | *r* = .044 | |
| Normalising beliefs (BAPS)^b^ | 16.14 (4.37) | 16.19 (4.43) | .868 | *r* = .014 | |
| Negative-self (BCSS)^b^ | 9.64 (5.03) | 7.19 (5.52) | .085 | *r* = .148 | |
| Positive-self (BCSS)^b^ | 4.93 (4.65) | 8.63 (4.77) | .005 | *r* = .239 | |
| Negative-other (BCSS)^b^ | 6.36 (4.48) | 5.93 (5.29) | .599 | *r* = .045 | |
| Positive-other (BCSS)^b^ | 6.21 (4.48) | 8.88 (4.72) | .040 | *r* = .177 | |
| Attachment anxiety (PAM)^b^ | 1.80 (0.61) | 1.65 (0.68) | .459 | *r* = .064 | |
| Attachment avoidance (PAM)^b^ | 2.18 (0.62) | 1.80 (0.50) | .007 | *r* = .232 | |
| Schizotypal cognitions (RISC)^b^ | 41.29 (11.01) | 37.92 (9.22) | .289 | *d* = 0.357 | |

ACEs, Adverse Childhood Experiences; BAPS, Beliefs About Paranoia Scale; BCSS, Brief Core Schema Scale; CHR-NT, clinical high-risk non-transitions; CHR-T, clinical high-risk transitions; GF, Global Functioning; IIP-32, Inventory of Interpersonal Problems; IPANAS-SF, International Positive and Negative Affect Schedule - Short Form; MINI, Mini-International Neuropsychiatric Interview; PAM, Psychosis Attachment Measure; RISC, Rust Inventory of Schizotypal Cognitions; SD, standard deviation; SIAS, Social Interaction Anxiety Scale; SOS, Significant Others Scale

^a^ Refers to n = 124 CHR-P participants (CHR-T = 14, CHR-NT = 110)

^b^ Refers to n = 135 CHR-P participants (CHR-T = 14, CHR-NT = 121)

^c^ Effect sizes were Rosenthal's *r* for Mann-Whitney U tests (small effect = .1, medium effect = .3, large effect = .5), Cohen’s *d* for Welch’s *t*-tests (small effect = 0.2, medium effect = 0.5, large effect = 0.8) and Phi (*ϕ*) for Pearson’s chi-squared or Fisher′s exact tests (small effect = .1, medium effect = .3, large effect = .5)

# Supplementary Table 12. Cumulative hazard rates for transition at each follow-up time point in the different risk criteria subgroups

| **Cumulative Hazard Rate** | | | | | | |
| --- | --- | --- | --- | --- | --- | --- |
| **Time (months)** | **6** | **12** | **18** | **24** | **30** | **36** |
| General Risk Criteria | | | | | | |
| SPI-A only | 0 | 0 | 0 | .06062 | .06062 | .06062 |
| CAARMS only | 0 | .03637 | .08082 | .14144 | .24680 | .24680 |
| Both | .05407 | .12003 | .12003 | .17560 | .17560 | .22689 |
| Specific Risk Criteria | | | | | | |
| APS & COGDIS | .07146 | .11798 | .11798 | .24314 | .24314 | .34850 |
| No APS & COGDIS | .01156 | .05028 | .06509 | .10283 | .13268 | .13268 |

APS, attenuated psychotic symptoms; CAARMS, Comprehensive Assessment of At-Risk Mental States; COGDIS, Cognitive Disturbances; SPI-A, Schizophrenia Proneness Instrument, Adult version

# Supplementary Table 13. Additional clinical, functional and questionnaire data for PFO (N = 67) versus GFO (N = 49) participants and persistent APS (N = 38) versus non-persistent APS (N = 47) participants at baseline

|  | **PFO**  **(N = 67)** | **GFO**  **(N = 49)** | ***p*** | **Effect size**^c^ | **Persistent APS**  **(N = 38)** | | **Non-persistent APS**  **(N = 47)** | | ***P*** | | **Effect size**^c^ | | |
| --- | --- | --- | --- | --- | --- | --- | --- | --- | --- | --- | --- | --- | --- |
| MINI data, n (%) | | | | | | | | | | | | | |
| Major depressive episode (past 2 weeks) | 31 (46.3) | 14 (28.6) | .053 | *ϕ* = .179 | | 16 (42.1) | | 19 (40.4) | | .876 | | *ϕ* = .017 | |
| Dysthymia (past 2 year) | 14 (20.9) | 4 (8.2) | .061 | *ϕ* = .174 | 6 (15.8) | | 7(14.9) | | .909 | | *ϕ* = .012 | |  |
| Suicide risk (past month) |  |  |  |  |  | |  | |  | |  | | |
| Low | 16 (23.9) | 8 (16.3) | .321 | *ϕ* = .092 | 7 (18.4) | | 12 (25.5) | | .434 | | *ϕ* = .085 | | |
| Medium | 11 (16.4) | 8 (16.3) | .990 | *ϕ* = .001 | 5 (13.2) | | 10 (21.3) | | .329 | | *ϕ* = .106 | | |
| High | 19 (28.4) | 2 (4.1) | < .001 | *ϕ* = .311 | 9 (23.7) | | 8 (17.0) | | .445 | | *ϕ* = .083 | | |
| (Hypo) manic episode (current) | 10 (14.9) | 5 (10.2) | .454 | *ϕ* = .070 | 4 (10.5) | | 6 (12.8) | | 1.000 | | *ϕ* = .035 | | |
| Panic disorder (past month) | 5 (7.5) | 3 (6.1) | 1.000 | *ϕ* = .026 | 2 (5.3) | | 5 (10.6) | | .453 | | *ϕ* = .097 | | |
| Agoraphobia (current) | 27 (40.3) | 17 (34.7) | .539 | *ϕ* = .057 | 15 (39.5) | | 18 (38.3) | | .912 | | *ϕ* = .012 | | |
| Social phobia (past month) | 27 (40.3) | 7 (14.3) | .002 | *ϕ* = .282 | 15 (39.5) | | 16 (34.0) | | .605 | | *ϕ* = .056 | | |
| Obsessive-compulsive disorder (past month) | 16 (23.9) | 2 (4.1) | .004 | *ϕ* = .270 | 10 (26.3) | | 4 (8.5) | | .028 | | *ϕ* = .239 | | |
| Posttraumatic stress disorder (past month) | 9 (13.4) | 1 (2.0) | .043 | *ϕ* = .200 | 5 (13.2) | | 4 (8.5) | | .505 | | *ϕ* = .075 | | |
| Alcohol dependence/abuse (past 12 months) | 21 (31.3) | 13 (26.5) | .574 | *ϕ* = .052 | 10 (26.3) | | 15 (31.9) | | .573 | | *ϕ* = .061 | | |
| Drug dependence/abuse (past 12 months) | 9 (13.4) | 8 (16.3) | .663 | *ϕ* = .040 | 5 (13.2) | | 8 (17.0) | | .623 | | *ϕ* = .053 | | |
| Anorexia nervosa (past 3 months) | 1 (1.5) | 0 (0) | 1.000 | *ϕ* = .080 | 1 (2.6) | | 0 (0) | | .447 | | *ϕ* = .121 | | |
| Bulimia nervosa (past 3 months) | 4 (6.0) | 4 (8.2) | .720 | *ϕ* = .043 | 4 (10.5) | | 4 (8.5) | | 1.000 | | *ϕ* = .034 | | |
| Generalised anxiety disorder (past 6 months) | 37 (55.2) | 19 (38.8) | .080 | *ϕ* = .163 | 22 (57.9) | | 23 (48.9) | | .411 | | *ϕ* = .089 | | |
| Functioning data, median (range) | | | | | | | | | | | | | |
| GF: Social lowest | 7 (3-9) | 7 (5-9) | .010 | *r* = .239 | 7 (3-8) | | 7 (4-9) | | .567 | | *r* = .062 | | |
| GF: Social highest | 8 (4-10) | 8 (7-10) | .031 | *r* = .201 | 8 (4-10) | | 8 (5-10) | | .695 | | *r* = .043 | | |
| GF: Role lowest | 7 (3-9) | 7 (5-9) | .002 | *r* = .284 | 7 (4-9) | | 7 (4-9) | | .310 | | *r* = .110 | | |
| GF: Role highest | 8 (3-9) | 8 (6-10) | .005 | *r* = .260 | 8 (5-9) | | 8 (4-9) | | .850 | | *r* = .020 | | |
| Questionnaire data, mean (SD) | | | | | | | | | | | | | |
| Positive affect (IPANAS-SF) | 24.55 (7.56)^a^ | 26.77 (6.03)^a^ | .084 | *d* = 0.319 | 24.19 (6.80)^b^ | | 25.07 (6.31)^b^ | | .548 | | *d* = 0.135 | | |
| Negative affect (IPANAS-SF) | 27.30 (7.51)^a^ | 24.69 (8.53)^a^ | .092 | *d* = 0.329 | 29.14 (8.83)^b^ | | 25.88 (7.87)^b^ | | .084 | | *d* = 0.392 | | |
| Interpersonal problems (IIP-32) | 1.60 (0.53)^a^ | 1.49 (0.49)^a^ | .278 | *d* = 0.204 | 1.56 (0.56)^b^ | | 1.59 (0.52)^b^ | | .782 | | *d* = 0.062 | | |
| Practical support satisfaction (SOS) | 0.98 (0.79)^a^ | 0.94 (0.64)^a^ | .984 | *r* = .002 | 0.96 (0.70)^b^ | | 0.99 (0.86)^b^ | | .843 | | *r* = .022 | | |
| Emotional support satisfaction (SOS) | 1.01 (0.79)^a^ | 1.13 (0.79)^a^ | .414 | *r* = .076 | 1.13 (0.76)^b^ | | 1.03 (0.80)^b^ | | .506 | | *r* = .073 | | |
| Social interaction anxiety (SIAS) | 41.57 (15.10)^a^ | 36.80 (12.60)^a^ | .066 | *r* = .172 | 41.46 (15.08)^b^ | | 39.35 (14.90)^b^ | | .526 | | *d* = 0.141 | | |
| Adverse Childhood Experiences (ACEs) | 2.45 (2.03) | 1.59 (1.74) | .012 | *r* = .234 | 2.37 (2.05) | | 2.06 (2.09) | | .406 | | *r* = .090 | | |
| Negative beliefs (BAPS) | 13.78 (4.41) | 13.17 (4.26) | .458 | *d* = 0.139 | 14.83 (3.91) | | 13.55 (4.64) | | .172 | | *d* = 0.295 | | |
| Survival beliefs (BAPS) | 11.60 (4.86) | 11.13 (3.67) | .935 | *r* = .008 | 11.55 (4.57) | | 11.47 (4.54) | | 1.000 | | *r* = .000 | | |
| Normalising beliefs (BAPS) | 16.33 (4.42) | 16.53 (4.42) | .693 | *r* = .037 | 16.16 (4.14) | | 16.00 (4.56) | | .865 | | *d* = 0.037 | | |
| Negative-self (BCSS) | 8.67 (6.11) | 5.77 (4.56) | .014 | *r* = .229 | 8.76 (6.11) | | 7.00 (5.49) | | .188 | | *r* = .143 | | |
| Positive-self (BCSS) | 7.61 (5.17) | 9.31 (4.60) | .054 | *r* = .179 | 8.24 (4.93) | | 7.78 (5.19) | | .681 | | *d* = 0.089 | | |
| Negative-other (BCSS) | 6.84 (5.40) | 4.37 (4.79) | .013 | *r* = .231 | 6.66 (5.57) | | 5.49 (5.26) | | .407 | | *r* = .090 | | |
| Positive-other (BCSS) | 8.31 (4.85) | 8.76 (4.67) | .630 | *r* = .045 | 8.66 (4.74) | | 7.91 (4.96) | | .482 | | *d* = 0.153 | | |
| Attachment anxiety (PAM) | 1.65 (0.60) | 1.63 (0.75) | .889 | *r* = .013 | 1.71 (0.68) | | 1.67 (0.67) | | .774 | | *d* = 0.063 | | |
| Attachment avoidance (PAM) | 1.95 (0.55) | 1.67 (0.46) | .004 | *d* = 0.542 | 1.93 (0.55) | | 1.84 (0.50) | | .414 | | *d* =0.181 | | |
| Schizotypal cognitions (RISC) | 39.21 (8.94) | 35.34 (10.07) | .035 | *d* = 0.410 | 40.47 (8.90) | | 37.55 (8.59) | | .131 | | *d* = 0.334 | | |

ACEs, Adverse Childhood Experiences; APS, attenuated psychotic symptoms; BAPS, Beliefs About Paranoia Scale; BCSS, Brief Core Schema Scale; GF, Global Functioning; GFO, good functional outcome; IIP-32, Inventory of Interpersonal Problems; IPANAS-SF, International Positive and Negative Affect Schedule - Short Form; MINI, Mini-International Neuropsychiatric Interview; PAM, Psychosis Attachment Measure; PFO, poor functional outcome; RISC, Rust Inventory of Schizotypal Cognitions; SD, standard deviation; SIAS, Social Interaction Anxiety Scale; SOS, Significant Others Scale

^a^ Refers to n = 114 CHR-P participants (PFO = 66, GFO = 48)

^b^ Refers to n = 83 CHR-P participants (Persistent APS = 37, Non-persistent APS = 46)

^c^ Effect sizes were Rosenthal's *r* for Mann-Whitney U tests (small effect = .1, medium effect = .3, large effect = .5), Cohen’s *d* for Welch’s *t*-tests (small effect = 0.2, medium effect = 0.5, large effect = 0.8) and Phi (*ϕ*) for Pearson’s chi-squared or Fisher′s exact tests (small effect = .1, medium effect = .3, large effect = .5)

**Baseline**

**CHR-P = 144 CHR-NT = 130**

- Demographics
- CAARMS positive scale
- SPI-A (COPER, COGDIS)
- GAF
- MINI^a^
- GF: Social^a^
- GF: Role^a^
- IIP-32^b^
- SOS^b^
- IPANAS-SF^b^

^a^N = 138 CHR-P ^b^N = 124 CHR-P

**6-12 months follow-up**

**CHR-P = 116 CHR-NT = 102**

**6 months**

- CAARMS positive scale
- SCID ⚫ GF: Role ⚫ GF: Social
- IIP-32 ⚫ SOS ⚫ IPANAS-SF

**CHR-T identified = 7**

**Dropouts since last follow-up = 28**

**12 months**

- CAARMS positive scale
- SCID ⚫ GF: Role ⚫ GF: Social
- IIP-32 ⚫ SOS ⚫ IPANAS-SF

**CHR-T identified = 2**

**Dropouts since last follow-up = 38**

**30-36 months follow-up**

**CHR-P = 45 CHR-NT = 43**

**18-24 months follow-up**

**CHR-P = 84 CHR-NT = 77**

**CHR-T identified = 5**

**Dropouts since last follow-up = 21**

**Missed follow-up = 4**

**30 months**

- CAARMS positive scale
- IIP-32 ⚫ SOS ⚫ IPANAS-SF

**18 months**

- CAARMS positive scale
- IIP-32 ⚫ SOS ⚫ IPANAS-SF

**24 months** ^c^N = 46 CHR-P

- CAARMS positive scale
- SPI-A (COPER, CODGIS)^c^
- SCID ⚫ GF: Role ⚫ GF: Social
- IIP-32 ⚫ SOS ⚫ IPANAS-SF

**36 months**

- CAARMS positive scale
- SPI-A (COPER, CODGIS)
- IIP-32 ⚫ SOS ⚫ IPANAS-SF

Supplementary Figure 1. CHR-P flowchart of baseline and follow-up assessments. Note that 4 CHR-NT completed the 30-36 months follow-up despite missing the 18-24 months follow-up. For CHR-P, the last available follow-up data is as follows: 6-12 months (6 months [n = 23]; 12 months [n=93]), 18-24 months (18 months [n = 20]; 24 months [n=64]) and 30-36 months (30 months [n = 6]; 36 months [n=39]). CAARMS, Comprehensive Assessment of At-Risk Mental States; CHR-P, clinical high-risk for psychosis; CHR-NT, clinical high-risk non-transitions; CHR-T clinical high-risk transitions; COGDIS, Cognitive Disturbances; COPER, Cognitive-Perceptive Basic Symptoms; GAF, Global Assessment of Functioning; GF, Global Functioning; IIP-32, Inventory of Interpersonal Problems; IPANAS-SF; International Positive and Negative Affect Schedule - Short Form; SCID, Structured Clinical Interview for DSM-IV; SOS, Significant Others Scale; SPI-A, Schizophrenia Proneness Instrument, Adult version.

**Baseline**

**CHR-N = 51**

- Demographics
- CAARMS positive scale
- SPI-A (COPER, COGDIS)
- GAF
- MINI^a^
- GF: Social^a^
- GF: Role^a^
- IIP-32^b^
- SOS^b^
- IPANAS-SF^b^

^a^N = 47 ^b^N = 42

**6-12 months follow-up**

**CHR-N = 28**

**6 months**

- CAARMS positive scale
- SCID ⚫ GF: Role ⚫ GF: Social
- IIP-32 ⚫ SOS ⚫ IPANAS-SF

**Met APS criteria = 6**

**Dropouts since last follow-up = 21**

**Missed follow-up = 2**

**12 months**

- CAARMS positive scale
- SCID ⚫ GF: Role ⚫ GF: Social
- IIP-32 ⚫ SOS ⚫ IPANAS-SF

**30-36 months follow-up**

**CHR-N = 18**

**18-24 months follow-up**

**CHR-N = 21**

**Met APS criteria = 3**

**Dropouts since last follow-up = 6**

**30 months**

- CAARMS positive scale
- IIP-32 ⚫ SOS ⚫ IPANAS-SF

**18 months**

- CAARMS positive scale
- IIP-32 ⚫ SOS ⚫ IPANAS-SF

**Met APS criteria = 4**

**Dropouts since last follow-up = 6**

**Missed follow-up = 3**

**24 months** ^c^N = 13

- CAARMS positive scale
- SPI-A (COPER, CODGIS)^c^
- SCID ⚫ GF: Role ⚫ GF: Social
- IIP-32 ⚫ SOS ⚫ IPANAS-SF

**36 months**

- CAARMS positive scale
- SPI-A (COPER, CODGIS)
- IIP-32 ⚫ SOS ⚫ IPANAS-SF

Supplementary Figure 2. CHR-N flowchart of baseline and follow-up assessments. Note that 1 CHR-N only completed the 18-24 months follow-up, 1 CHR-N only completed the 30-36 months follow-up, and 2 CHR-N completed the 30-36 months follow-up despite missing the 18-24 months follow-up. For CHR-N, the last available follow-up data is as follows: 6-12 months (6 months [n = 1]; 12 months [n=27]), 18-24 months (18 months [n = 5]; 24 months [n=16]) and 30-36 months (30 months [n = 5]; 36 months [n=13]). APS, attenuated psychotic symptoms; CAARMS, Comprehensive Assessment of At-Risk Mental States; CHR-N, clinical high-risk-negative; COGDIS, Cognitive Disturbances; COPER, Cognitive-Perceptive Basic Symptoms; GAF, Global Assessment of Functioning; GF, Global Functioning. IIP-32, Inventory of Interpersonal Problems; IPANAS-SF; International Positive and Negative Affect Schedule - Short Form; SCID, Structured Clinical Interview for DSM-IV; SOS, Significant Others Scale; SPI-A, Schizophrenia Proneness Instrument, Adult version
